# Global Transcriptomics Uncovers Distinct Contributions From Splicing Regulatory Proteins to the Macrophage Innate Immune Response

**DOI:** 10.3389/fimmu.2021.656885

**Published:** 2021-07-09

**Authors:** Allison R. Wagner, Haley M. Scott, Kelsi O. West, Krystal J. Vail, Timothy C. Fitzsimons, Aja K. Coleman, Kaitlyn E. Carter, Robert O. Watson, Kristin L. Patrick

**Affiliations:** ^1^ Department of Microbial Pathogenesis and Immunology, College of Medicine, Texas A&M Health, Bryan, TX, United States; ^2^ Department of Veterinary Pathobiology, Texas A&M University, College Station, TX, United States

**Keywords:** pre-mRNA splicing, RNA binding protein, inflammation, hnRNP, SR protein, *Salmonella* Typhimurium

## Abstract

Pathogen sensing *via* pattern recognition receptors triggers massive reprogramming of macrophage gene expression. While the signaling cascades and transcription factors that activate these responses are well-known, the role of post-transcriptional RNA processing in modulating innate immune gene expression remains understudied. Given their crucial role in regulating pre-mRNA splicing and other RNA processing steps, we hypothesized that members of the SR/hnRNP protein families regulate innate immune gene expression in distinct ways. We analyzed steady state gene expression and alternatively spliced isoform production in ten SR/hnRNP knockdown RAW 264.7 macrophage-like cell lines following infection with the bacterial pathogen *Salmonella enterica* serovar Typhimurium (*Salmonella*). We identified thousands of transcripts whose abundance is increased or decreased by SR/hnRNP knockdown in macrophages. Notably, we observed that SR and hnRNP proteins influence expression of different genes in uninfected versus *Salmonella*-infected macrophages, suggesting functionalization of these proteins upon pathogen sensing. Likewise, we found that knockdown of SR/hnRNPs promoted differential isoform usage (DIU) for thousands of macrophage transcripts and that these alternative splicing changes were distinct in uninfected and *Salmonella*-infected macrophages. Finally, having observed a surprising degree of similarity between the differentially expressed genes (DEGs) and DIUs in hnRNP K and U knockdown macrophages, we found that hnRNP K and U knockdown macrophages are both more restrictive to Vesicular Stomatitis Virus (VSV), while hnRNP K knockdown macrophages are more permissive to *Salmonella* Typhimurium. Based on these findings, we conclude that many innate immune genes evolved to rely on one or more SR/hnRNPs to ensure the proper magnitude of their induction, supporting a model wherein pre-mRNA splicing is critical for regulating innate immune gene expression and controlling infection outcomes in macrophages *ex vivo*.

## Introduction

When innate immune cells like macrophages sense pathogens, they undergo dramatic gene expression reprogramming and upregulate thousands of genes. Proper regulation of the timing and magnitude of innate immune gene induction is critical to ensure that the immune system is adequately stimulated to fend off microbial invaders without risking deleterious outcomes associated with hyperinflammation (1–3). While there has been great interest in the mechanisms of pathogen sensing and signaling events that activate transcription following an inflammatory signal, much less is known about how RNA processing steps downstream of transcription influence innate immune gene expression outcomes.

Consistent with the current “transcription-focused” paradigm of innate immune gene expression, research has categorized innate immune genes into primary and secondary response genes (4, 5). Primary, or early response genes, are readily induced upon activation of pathogen sensing cascades. Many of these transcripts reach maximal abundance as soon as 30 minutes post-pathogen sensing (6, 7). Secondary response genes require the activation of a transcription factor or expression of a cytokine before they can be maximally induced. The timing and induction of primary and secondary response genes relies on a number of tightly regulated mechanisms, including but not limited to, cooperative binding of transcription factors (8, 9), nucleosome occupancy and histone modification at promoters (10, 11), signal-dependent interactions between transcription factor subunits (12, 13), and selective interaction with the transcriptional elongation machinery (14).

Following this carefully orchestrated transcriptional activation, innate immune transcripts, like most eukaryotic transcripts, are subject to post-transcriptional regulation at the level of pre-mRNA splicing, cleavage and polyadenylation, mRNA export, and nonsense mediated decay. Pre-mRNA splicing is increasingly appreciated as an important regulatory node in cells undergoing stress or responding to extracellular triggers, including exposure to vitamins and metal ions (15), heat shock (16–18), and UV damage (19, 20). Specifically, there is growing interest in how RNA processing modulates innate immune gene expression and infection outcomes. Both *Salmonella enterica* and *Listeria monocytogenes* infection promote widespread 3’UTR shortening and exon inclusion in primary human macrophages (21) and alternative splicing and nonsense mediated decay play important roles in balancing isoform abundance of key antiviral innate immune molecules like *Oas1g* (22). Important kinetic studies of gene expression in Lipid A (a component of lipopolysaccharide)-treated primary murine macrophages showed that intron removal and release of processed innate immune transcripts from chromatin can be significantly delayed relative to onset of a gene’s transcription (6, 7). While these findings argue that post-transcriptional regulatory mechanisms play a key role in controlling the timing and abundance of translation-competent immune mRNAs, we still know very little about the mechanisms that drive this regulation and the specific macrophage factors involved.

Extracellular signal transduction provides one potential mechanism through which splicing factors may be regulated during the innate immune response. Several studies have demonstrated that differential phosphorylation of SR family members triggers distinct splicing changes in cells responding to heat-shock (16–18). SR (serine-arginine rich) proteins direct the spliceosome to particular regions of a pre-mRNA by binding conserved sequences called exonic splicing enhancers or silencers. SR proteins are considered “activators” of gene expression and generally promote exon inclusion. Conversely, heterogenous nuclear ribonucleoproteins (hnRNPs) typically work to repress splicing by binding conserved sequences within introns. SR and hnRNP proteins often work cooperatively and antagonistically to control pre-mRNA splicing decisions. Recent global phosphoproteomics studies revealed that proteins involved in mRNA processing, including a number of SR and hnRNPs, are among the most differentially phosphorylated proteins in macrophages following infection with a bacterial (23, 24) or fungal pathogen (25). These findings motivated our interest to identify gene expression and alternative splicing changes dictated by SR and hnRNP family members in macrophages and to compare how these events change following infection with a bacterial pathogen.

To begin investigating how splicing regulatory proteins dictate gene expression and alternative splicing changes during the macrophage innate immune response, we took an unbiased approach and knocked down expression of ten members of the SR/hnRNP families of splicing regulatory factors. We infected these knockdown cell lines with *Salmonella enterica* serovar Typhimurium (*Salmonella*) and measured differentially expressed genes (DEGs) and differential isoform usage (DIU) in steady state RNA at a key early innate immune time point (4h post-infection). Our analysis found that these SR/hnRNPs regulate the abundance or splicing of many different cohorts of genes. Curiously, genes whose abundance changed in SR/hnRNP knockdowns (DEGs) were not also subject to differential isoform usage. While the reliance of innate immune transcripts on SR/hnRNPs did not correlate with induction level, gene length, or exon/intron number, we did observe that many primary response genes are hyperinduced in *Salmonella*-infected SR/hnRNP knockdown macrophages, suggesting a role for splicing regulatory proteins in repressing the early innate immune response. Together, our data implicate splicing proteins in fine-tuning the magnitude of innate immune gene induction and highlight an underappreciated role for RNA binding proteins in controlling intracellular infection outcomes.

## Materials and Methods

### Cell Lines and Bacterial Strains

RAW 264.7 macrophages (ATCC) (originally isolated from male BALB/c mice) were cultured at 37°C with a humidified atmosphere of 5% CO_2_ in DMEM (Thermo Fisher) with 10% FBS (Sigma Aldrich) 0.5% HEPES (Thermo Fisher). For knockdown cell lines, RAW 264.7 macrophages were transduced with a pSICO-shRNA construct designed to target an exon or 3’UTR of an SR or hnRNP gene of interest. Knockdown macrophages were drug selected (hygromycin; Invitrogen) alongside a scramble (SCR) untargeted control. Each SR knockdown cell line was derived at the same time, as were the hnRNP cell lines. Knockdown efficiency of each factor was validated by RT-qPCR using exonic primer sets and the most efficient knockdown cell line (from 6 different knockdown constructs) was used for RNA-seq. The two most efficient knockdown cell lines were used for validation in **Figures 3** and **4**.

### *S*. Typhimurium Infections

Infections with *Salmonella enterica* serovar Typhimurium were conducted by plating RAW 264.7 macrophages on tissue-cultured treated 12-well dishes at 7.5 x10^5^ and incubated overnight. Overnight cultures of *S*. Typhimurium were diluted 1:20 in LB broth containing 0.3M NaCl and grown until they reached an OD600 of 0.9. Unless specified, cell lines at a confluency of 80% were infected with the *S*. Typhimurium strains at an MOI (multiplicity of infection) of 10 for 30 minutes in Hank’s buffered salt solution (HBSS). Infected monolayers were spun for 10 minutes at 1,000rpm, washed twice in HBSS containing 100μg/ml of gentamycin, and refreshed with media plus gentamicin (10 μg/ml). After removal of supernatant, cells were lysed in Trizol (Thermo Fisher) for RNA collection and cDNA was analyzed using RT-qPCR. For colony forming units (CFUs), RAW 264.7 macrophages were plated on tissue-cultured treated 24-well dishes at 5 x10^5^. Overnight cultures of *S*. Typhimurium were diluted to OD600 of 1.0 and cell lines at a confluency of 80% were infected at an MOI of 10 (as above). After removal of supernatant, cells were washed 2X in Phosphate-Buffered Saline (PBS). Cell were lysed in 1ml of PBS+1%TritonX100 + 0.01%SDS. Serial dilutions of the lysed cells were made in PBS and plated in duplicate on LB plates and incubated at 37°C overnight.

### RNA-Seq

The RNA-Seq experiment was made up of 60 samples: biological triplicate of SCR uninfected, SCR *Salmonella*-infected, each knockdown uninfected, and each *Salmonella*-infected knockdown. RNA-Seq and library preparation was performed by Texas A&M AgriLife Genomics and Bioinformatics Service. Samples were sequenced on Illumina 4000 using 2 × 150-bp paired-end reads. Raw reads were filtered and trimmed and Fastq data was mapped to the Mus musculus Reference genome (RefSeq) using CLC Genomics Workbench 8.0.1. Differential expression analyses were performed using CLC Genomics Workbench. Relative transcript expression was calculated by counting Reads Per Kilobase of exon model per Million mapped reads (RPKM). statistical significance was determined by the EDGE test *via* CLC Genomics Workbench. Differentially expressed genes (DEGs) were selected as those with p value threshold < 0.05.

### Gene Ontology (GO) Ingenuity Pathway Analysis and Hierarchical Clustering

To determine the most affected pathways in control versus knockdown RAW 264.7 macrophages, canonical pathway analysis was conducted using Ingenuity Pathway Analysis software from QIAGEN Bioinformatics. Genes that were differentially expressed with a p value < 0.05 from our RNA-SEQ analysis were used as input from uninfected and *Salmonella* Typhimurium infected cells. Hierarchical clustering was done in Cluster3 (3.0) with complete linkage, absolute correlation (centered) parameters and visualized using Java TreeView.

### Scatter Plots and Correlation Analysis

For (p<0.05) differentially expressed genes, fold change was plotted to compare to coding sequence length which is identified by CLC Genomics Workbench to be equal to the total length of all exons (not all transcripts). Exon number and intron number were identified by CLC Genomics Workbench to be the number of exons/introns based on the mRNA annotations of the reference genome. Total gene length was calculated using “chromosome region start” and “chromosome region end” which are determined by CLC Genomics Workbench and the reference sequence to be the start position and end position of the annotated gene. Pearson Correlation was calculated using the values described above.

### RNA Isolation and RT-qPCR Analysis

For transcript analysis, cells were harvested in Trizol and RNA was isolated using Direct-zol RNA Miniprep kits (Zymo Research) with 1 hr DNase treatment. cDNA was synthesized with iScript cDNA Synthesis Kit (Bio-Rad). RT-qPCR was performed using Power-Up SYBR Green Master Mix (Thermo Fisher) using a Quant Studio Flex 6 (Applied Biosystems). Averages of the raw values were normalized to average values for the same sample with the control gene, *Actb*. To analyze fold induction, the average of the treated sample was divided by the untreated SCR control sample, which was set at 1.

### Alternative Splicing Analysis

Alternative splicing events were analyzed using Modeling Alternative Junction Inclusion Quantification (MAJIQ) and VOILA (a visualization package) with the default parameters (26). Briefly, uniquely mapped, junction-spanning reads were used by MAJIQ to construct splice graphs for transcripts by using the RefSeq annotation supplemented with de-novo detected junctions. Here, de-novo refers to junctions that were not in the RefSeq transcriptome database but had sufficient evidence in the RNA-Seq data. The resulting gene splice graphs were analyzed for all identified local splice variations (LSVs). For every junction in each LSV, MAJIQ then quantified expected percent spliced in (PSI) value in control and knockdown samples and expected change in PSI (dPSI) between control and knockdown samples. Results from VOILA were then filtered for high confidence changing LSVs (whereby one or more junctions had at least a 95% probability of expected dPSI of at least an absolute value of 10 PSI units between control and knockdown) and candidate changing LSVs (95% probability, 10% dPSI). For these high confidence results (ΔPSI 10%), the events were further categorized as single exon cassette, multi-exon cassette, alternative 5′ and/or 3′ splice site, or intron-retention.

### RBP Finder

For each gene, the canonical (longest) isoform of the gene (5’ and 3’ UTRs, plus CDS) as annotated by Ensembl [Mouse (GRCm38.p6)] was queried for SR/hnRNP motifs as defined by RBPmap. Stringency level was set on “High” and the Conservation Filter was applied. In cases where multiple motifs were listed, only a single “consensus” motif was chosen (27).

### VSV Infection

7x10^5^ RAW cells were seeded in 12-well plates 16h before infection. Cells were infected with VSV-GFP virus at multiplicity of infection (MOI) of 1 in serum-free DMEM (HyClone SH30022.01). After 1h of incubation with media containing virus, supernatant was removed, and fresh DMEM plus 10% FBS was added to each well. At indicated times post infection, cells were harvested with Trizol and prepared for RNA isolation.

### Quantitation and Statistical Analysis

Statistical analysis of data was performed using GraphPad Prism software. Two-tailed unpaired Student’s t tests were used for statistical analyses, and unless otherwise noted, all results are representative of at least three biological experiments [mean ± STDEV (n = 3 per group)].

## Results

### SR and hnRNPs Regulate the Abundance of Distinct Sets of Transcripts in Uninfected and *Salmonella*-Infected Macrophages

**Figure 1:**
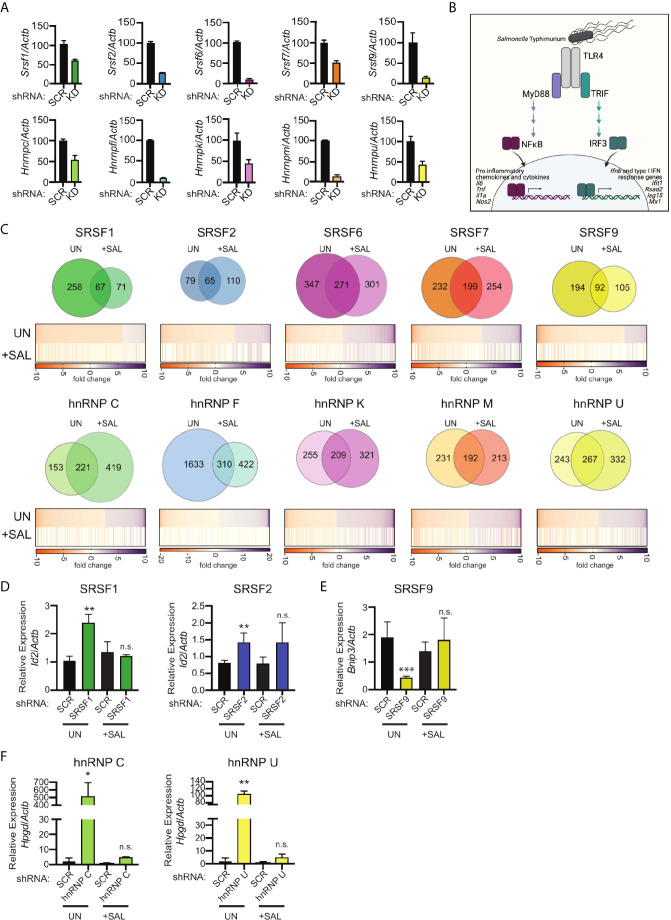
RNA-Seq reveals distinct hnRNP- and SRSF-dependent regulons in uninfected and *Salmonella*-infected RAW 264.7 macrophages. **(A)** Knockdown efficiency for each SR and hnRNP factor as measured by RT-qPCR. Data is shown as hnRNP/SRSF expression, relative to *Actb*, compared to SCR control cells. Ratios are the mean of 3 biological replicates and error bars show standard deviation. **(B)** Schematic representation of transcription factor activation downstream of *Salmonella* Typhimurium sensing by TLR4. **(C)** (Top) Overlap of differentially expressed genes (DEGs) between uninfected and *Salmonella*-infected RAW 264.7 macrophages (4h post-infection; MOI = 10) *via* Venn Diagram. (Bottom) Heatmaps of up and down-regulated DEGs from uninfected macrophages (UN). The values of the same genes in *Salmonella*-infected macrophages are shown below, with “blank spots” indicating DEGs that are not significantly changed in *Salmonella*-infected SR/hnRNP knockdown cell lines (+SAL). Orange represents genes downregulated in knockdown vs. SCR; purple represents genes upregulated in knockdown vs. SCR (colorbar shown below). DEGs were defined as having a statistically significant fold-change relative to SCR; p<0.05. **(D)** Relative gene expression of *Id2* over *Actb* in uninfected (UN) and *Salmonella*-infected (+SAL) SRSF1 and SRSF2 knockdown macrophage cell lines. **(E)** As in **(D)** but for *Bnip3* expression in SRSF9 knockdown macrophages. **(F)** As in **(D)** but for *Hpgd* in hnRNP C and hnRNP U knockdown macrophages. For D-F RT-qPCRs, values are the mean of 3 biological replicates and error bars indicate standard deviation. *p < 0.05; **p < 0.01; ***p < 0.001; n.s, not statistically significant (p > 0.05). **(B)** was created using Biorender.

To understand how splicing regulatory proteins shape global innate immune gene expression, we prioritized factors most likely to play a privileged role in the macrophage innate immune response. Two recent publications identified a number of splicing factors that were differentially phosphorylated during infection with the intracellular bacterial pathogen *Mycobacterium tuberculosis* in a murine macrophage-like cell line (RAW 264.7) (24) or in primary mouse macrophages (23). Based on these proteomics data (**Figure S1A**), we selected SRSF1, SRSF2, SRSF6, SRSF7, SRSF9, hnRNP C, hnRNP F, hnRNP K, hnRNP M, and hnRNP U for transcriptomics analysis. To begin, we generated RAW 264.7 cell lines in which each factor was stably knocked down *via* expression of an shRNA construct targeting an exon or the 3’UTR for each factor, with regions chosen to ensure that all protein coding isoforms of each factor would be targeted by the shRNA. Overall, six shRNA hairpins were tested for each SR/hnRNP and the two cell lines with the best knockdown efficiency were chosen for subsequent analysis. Interestingly, overall knockdown efficiency varied between factors, with only about 50% knockdown efficiency achieved for hnRNP C, hnRNP K, hnRNP U, SRSF1 and SRSF7 and 70-90% knockdown achieved for SRSF2, SRSF6, SRSF9, hnRNP F and hnRNP M (**Figure 1A**). We predict that variation in knockdown efficiency reflects the macrophage’s ability to tolerate loss of each of these factors and likely correlates with the cell’s reliance on each for maturation of essential housekeeping genes. The major risk of incomplete knockdown is missing phenotypes (false negatives), as opposed to reporting a false positive phenotype. Therefore, we concluded that these knockdown cell lines were sufficient to identify SR/hnRNP-sensitive innate immune genes and carried out our analysis with the caveat of differential knockdown efficiency in mind.

To induce macrophage innate immune gene expression, we infected each of the RAW 264.7 knockdown cell lines alongside two scramble shRNA hairpin-expressing (SCR) control cell lines with *Salmonella enterica* serovar Typhimurium at an MOI of 10. *Salmonella* is a gram-negative bacterium that triggers TLR4 sensing of *Salmonella* lipopolysaccharide (LPS). TLR4 signaling is unique amongst TLRs in that it activates two major innate immune transcription factor regulons: NFκB downstream of the MyD88 adapter protein, which activates expression of many pro-inflammatory cytokines and chemokines, and IRF3 downstream of the adapter TRIF (28), which turns on a type I interferon response characterized by *Ifnb* and interferon stimulated gene (ISG) expression (**Figure 1B**). We predicted that *Salmonella* infection, which triggers a physiologically-relevant macrophage response, would enable appreciation of even subtle contributions of SR/hnRNPs, while still allowing comparison between our findings and previous studies that focused on the dynamics of NFκB/IRF3 gene expression following direct delivery of LPS (6, 29). We collected total RNA from uninfected and *Salmonella*-infected macrophages at 4h post-infection and performed bulk RNA sequencing *via* an Illumina HiSeq 4000 sequencer (150bp; paired-end reads). An average of ~60.2 million raw sequencing reads were generated from three biological replicates (20 million reads per sample) of each knockdown (both in uninfected and *Salmonella*-infected conditions).

To determine if knockdown of SR and hnRNP proteins affected expression of different transcripts in uninfected vs. *Salmonella*-infected macrophages, we first identified transcripts whose expression was significantly altered (p<0.05; up- or down-regulated) in knockdown cell lines relative to controls. We deemed these “Differentially Expressed Genes” or DEGs. Venn diagrams were generated to visualize differences and overlap between affected genes in uninfected (UN) and Salmonella-infected (+SAL) macrophages (**Figure 1C** and **Supplementary Table 1**). About 1/3 of DEGs had altered expression in both uninfected and *Salmonella*-infected splicing factor knockdown cell lines (compared to SCR controls). This means that in the absence of any single splicing factor queried, about two-thirds of DEGs are unique to either condition (UN or +SAL) (**Figure 1C**, Venn diagrams). On average, expression of between 200-400 genes was altered in an SR or hnRNP knockdown cell line in either condition at this 4h time point. One notable exception, hnRNP F knockdown, altered the abundance of 1943 genes in uninfected macrophages and 732 genes in *Salmonella*-infected macrophages (**Supplementary Table 1** contains all gene expression changes p<0.05). It is possible that the strength of this phenotype could be due in part to the high knockdown efficiency of hnRNP F (**Figure 1A**).

As expected, many of the DEGs in *Salmonella*-infected cells were not represented amongst the uninfected DEGs (**Figure 1C**, Venn Diagrams). Most innate immune genes including cytokines, chemokines, and antimicrobial mediators are dramatically upregulated upon pathogen sensing and these genes are either expressed at very low levels or not at all in uninfected macrophages. However, we were surprised to find that many genes involved in basic cellular homeostasis and metabolism whose expression was impacted by loss of SR/hnRNPs in uninfected (UN) macrophages were not as impacted in the context of *Salmonella* infection (+SAL) (**Figure 1C**, heatmap comparisons).

To begin to understand why many housekeeping genes are sensitive to loss of SR/hnRNPs in uninfected but not *Salmonella*-infected macrophages, we cross-referenced uninfected SR/hnRNP-sensitive genes against all downregulated genes in SCR control macrophages at 4h-post *Salmonella*-infection (**Supplementary Table 1**). We found that 365 genes were downregulated 2-fold or more (p<0.05) in SCR control macrophages at 4h post-*Salmonella* infection (**Supplementary Table 1**). Many of these genes (e.g. *Lhfpl2*, *Bhlhe41*, *Hyal1*, and *Tbc1d2*) have previously been reported as differentially expressed in M1 vs. M2 macrophages and their downregulation likely represents M1 polarization that occurs following *Salmonella* infection (30). Surprisingly, only a handful of these downregulated genes were among the SR/hnRNP-sensitive genes in uninfected macrophages (**Figure S1B**). To directly test whether SR/hnRNP-sensitive genes in uninfected macrophages are less abundant in *Salmonella*-infected cells, we performed RT-qPCR on a set of genes (*Bnip3*, *Id2*, *Hpgd*), which encode proteins involved in regulating cell death, transcription, and prostaglandin metabolism. Consistent with our RNA-seq data, we observed SR/hnRNP-dependent changes in *Bnip3*, *Id2*, and *Hpgd* abundance only in uninfected macrophages (**Figures 1D–F**, UN). We measured no detectable change in the abundance of these transcripts in uninfected vs. infected SCR control macrophages. Overall, we observed that no more than 6.3% of uninfected DEGs were downregulated upon *Salmonella* infection (**Figure S1B**), supporting our initial hypothesis that SR proteins and hnRNPs are functionalized such that they influence expression of distinct genes in uninfected and *Salmonella*-infected macrophages, and this includes genes that are constitutively expressed in both conditions.

### SR and hnRNPs Contribute to Activation and Repression of Genes in Innate Immune-Related Pathways in *Salmonella*-Infected Macrophages

**Figure 2:**
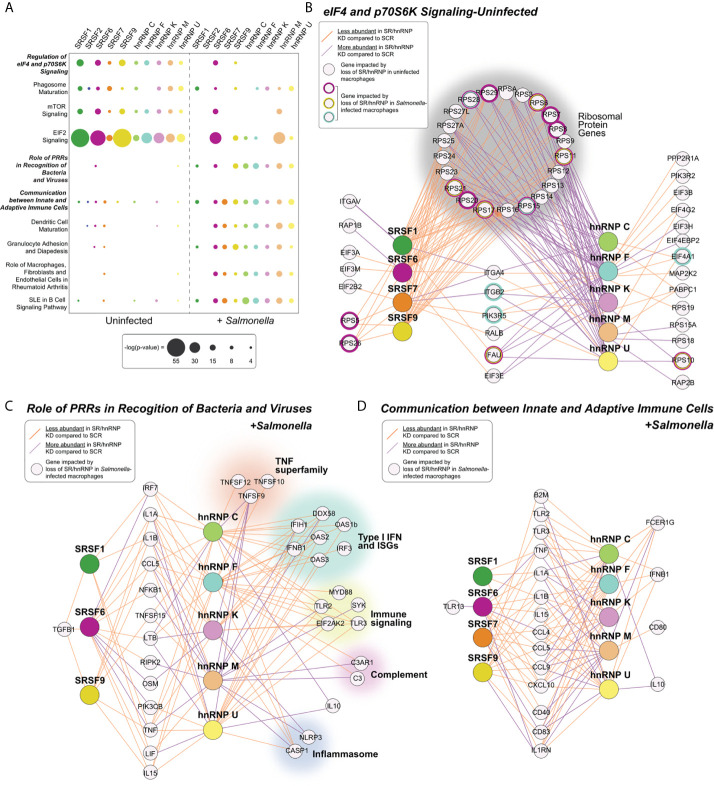
Pathways enriched for SR/hnRNP-dependent DEGs differ between uninfected and *Salmonella*-infected macrophages. **(A)** Canonical Ingenuity pathway analysis (IPA) of functional cellular pathways enriched for differentially expressed genes (DEGs) in uninfected (UN) and *Salmonella*-infected (+SAL) SR and hnRNP knockdown RAW 264.7 macrophages. Pathways enriched in eight or more knockdown cell lines in at least one condition are shown. Statistical enrichment is expressed as [-log (p-value)]. **(B)** Network diagrams showing DEGs from the IPA category “eIF4 and p70S6K Signaling” from each SR/hnRNP knockdown cell line in uninfected and *Salmonella* infected RAW 264.7 macrophages. Only SR/hnRNPs that showed DEG enrichment for the eIF4 pathway are shown. **(C)** As in **(B)** but for DEGs in *Salmonella*-infected SR/hnRNP knockdown cell lines in the IPA category “Role of Pattern Recognition Receptors in Recognition of Bacteria and Viruses.” **(D)** As in **(B)** but for the IPA category “Communication between Innate and Adaptive Immune Cells.” Purple lines connect SR or hnRNPs with target genes whose expression is upregulated in knockdown vs. SCR control macrophages. Orange lines connect SR or hnRNPs with target genes whose expression is downregulated in knockdown vs. SCR control macrophages. Cut-off for inclusion in the IPA was p < 0.05 for differential expression between knockdown and SCR cells.

As another measure of how SR/hnRNPs differentially influence gene expression in uninfected vs. *Salmonella*-infected macrophages, we performed Ingenuity Pathway Analysis (IPA) (Qiagen) to identify pathways enriched for SR/hnRNP-sensitive DEGs. In uninfected macrophages, we observed significant enrichment for DEGs in pathways related to translation initiation, mTOR signaling, and phagosomal maturation (**Figure 2A**, uninfected and **Supplementary Table 1** for full list). This finding is consistent with our previous analysis of hnRNP M-sensitive genes in uninfected macrophages (31) and the well-characterized role for splicing in controlling translation outcomes *via* ribosomal protein gene processing (32–34). We also performed IPA for the aforementioned 365 genes that are downregulated (>2-fold down) upon *Salmonella* infection in control macrophages described above and saw no overlap between these pathways and those enriched for SR/hnRNP-sensitive DEGs (**Figure S2A**). This too supports our conclusion that SR/hnRNP regulated genes are not globally downregulated upon infection.

Major pathways enriched for SR/hnRNP DEGs in *Salmonella*-infected macrophages are generally related to innate immune responses and macrophage activation, including “Role of Pattern Recognition Receptors (PRRs) in Recognition of Bacteria and Viruses,” “Communication between Innate and Adaptive Immune Cells”, and “Granulocyte Adhesion and Diapedesis” (**Figure 2A**, +*Salmonella*). Consistent with our observation that many DEGs identified in uninfected macrophages “lose reliance” on SR/hnRNPs upon *Salmonella*-infection, the only pathway that was significantly enriched for DEGs in both conditions was the translation/mTOR related pathway “Regulation of eIF4 p70S6K,” which remained significantly enriched for DEGs in SRSF6, SRSF9, and hnRNP F in *Salmonella*-infected macrophages (**Figure 2B**). We observed that while many transcripts had altered abundance in both SR and hnRNP knockdown macrophages, loss of SRs generally led to lower abundance (orange lines) while loss of hnRNPs led to higher abundance (purple lines). This same trend was evident for genes in innate immune pathways (**Figures 2C, D**). Interestingly, this gene-level analysis highlighted hnRNP-specific regulation of a diverse set of critical immune genes, including the potent anti-inflammatory mediator IL-10, members of the TNF superfamily (*Tnfsf12*, *Tnfsf10*, *Tnfsf9*), factors involved in the type I interferon response (*Ddx58* (RIG-I), *Ifih1*, *Oas1b*, *Oas2*, *Ifnb1*, *Oas3*, *Irf3*), as well as components of the complement cascade (*C3*, *C3ar1*) and the inflammasome (*Casp1*, *Nlrp3*) (**Figure 2C**). Together, these analyses suggest that proper gene expression levels in macrophages are maintained by balancing the activities of activating SR proteins and repressive hnRNPs.

**Figure 3:**
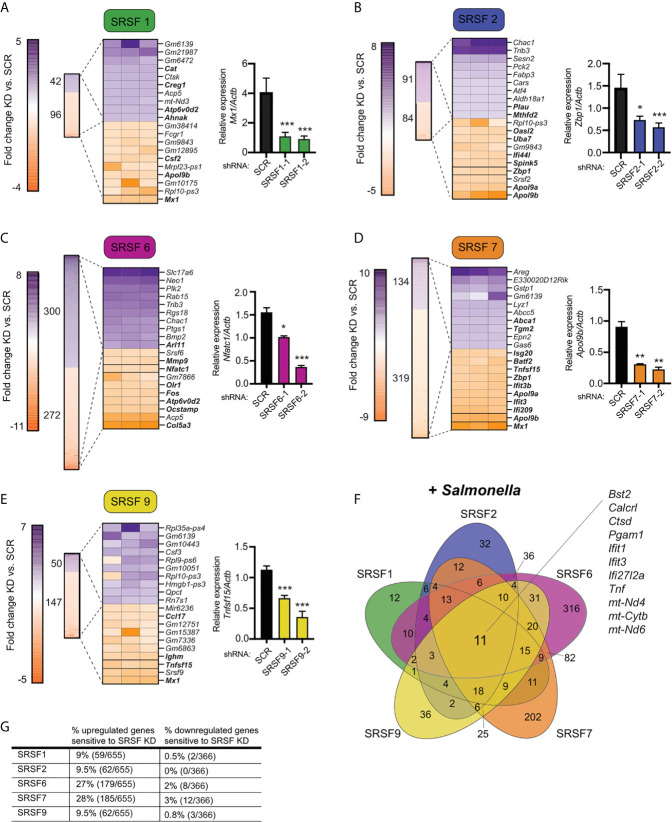
Knockdown of SR family members causes both up- and down-regulation of gene expression in *Salmonella*-infected macrophages. **(A)** On left, heatmap represents all up- and down-regulated genes in SRSF1 knockdown RAW 264.7 macrophages relative to SCR control at 4h post-*Salmonella* infection (p<0.05). Numbers next to heatmap indicate the total number of up- (purple) or down- (orange) regulated genes in SRSF1 knockdown cell lines vs. SCR. Zoom in represents the top 10 up- and down-regulated genes. Genes whose expression is up- or down-regulated by virtue of *Salmonella*-infection itself (i.e. innate immune regulated genes) according to analysis of *Salmonella*-infected SCR vs. uninfected SCR cells are bolded. Box indicates gene chosen for RT-qPCR validation. On right, RT-qPCR validation of a representative DEG (*Mx1*) in two SRSF1 knockdown cell lines vs. SCR control cells (data shown relative to *Actb*). **(B)** As in **(A)** but for SRSF2; RT-qPCR of *Zbp1*. **(C)** As in (A) but for SRSF6; RT-qPCR of *Nfatc1*; **(D)** As in **(A)** but for SRSF7; RT-qPCR of *Apol9b;* **(E)** As in **(A)** but for SRSF9; RT-qPCR of *Tnfsf15*. **(F)** Venn diagram of DEGs common to one or more SR knockdown cell line (p<0.05). The 11 genes whose expression is impacted by loss of all five SRSF proteins are highlighted. **(G)** Percentage of all genes induced at 4h post-*Salmonella* infection (>2.0-fold) that are differentially expressed in each SRSF knockdown macrophage cell line (p<0.05). For all RT-qPCRs, values are the mean of 2 or 3 biological replicates and error bars indicate standard deviation. *p < 0.05; **p < 0.01; ***p < 0.001.

**Figure 4:**
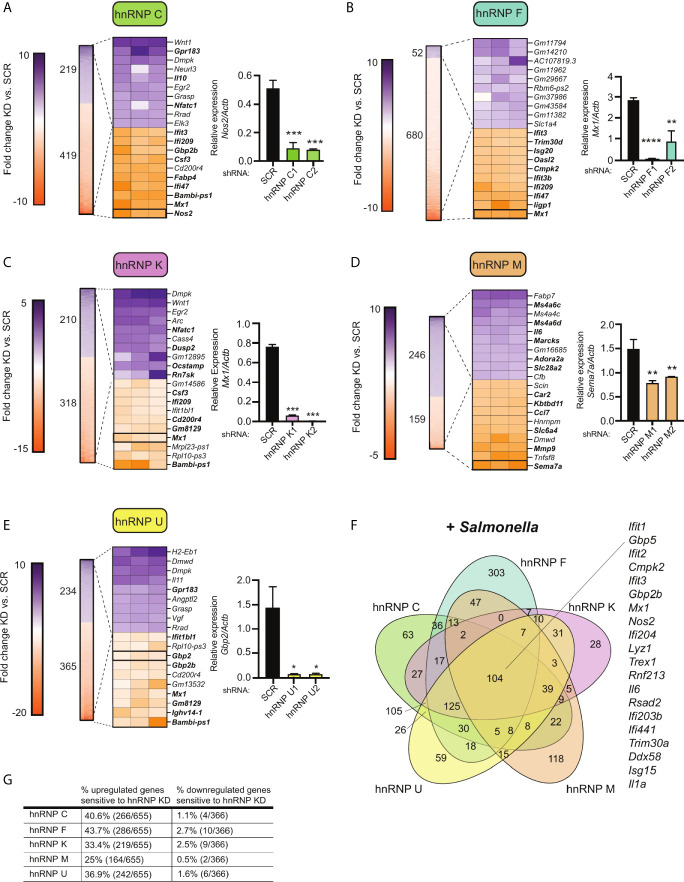
Knockdown of hnRNP family members causes up- and down-regulation of gene expression in *Salmonella*-infected macrophages. **(A)** On left, heatmap represents all up- and down-regulated genes in hnRNP C knockdown macrophages relative to SCR control at 4h post-*Salmonella* infection (p<0.05). Numbers next to heatmap indicate the total number of up- (purple) or down- (orange) regulated genes in hnRNP C knockdown cell lines vs. SCR. Zoom in represents the top 10 up- and down-regulated genes. Genes whose expression is up- or down-regulated by virtue of *Salmonella*-infection itself (i.e. innate immune regulated genes) according to analysis of *Salmonella*-infected SCR vs. uninfected SCR cells are bolded. Box indicates gene chosen for RT-qPCR validation. On right, RT-qPCR validation of a representative DEG (*Nos2*) in two knockdown cell lines vs. a SCR control (data shown relative to *Actb*). **(B)** As in **(A)** but for hnRNP F; RT-qPCR of *Mx1*. **(C)** As in **(A)** but for hnRNP K; RT-qPCR of *Mx1*. **(D)** As in **(A)** but for hnRNP M; RT-qPCR of *Sema7*; **(E)** As in **(A)** but for hnRNP U; RT-qPCR of *Gbp2*; **(F)** Venn diagram of DEGs common to one or more hnRNP knockdown cell line (p<0.05). A subset of the 104 genes whose expression was impacted by loss of all five hnRNP proteins are highlighted. **(G)** Percentage of all genes induced at 4h post-*Salmonella* infection (>2.0-fold) that are differentially expressed in each hnRNP knockdown macrophage cell line (p<0.05). For all RT-qPCRs values are the mean of 2 or 3 biological replicates and error bars indicate standard deviation. *p < 0.05; **p < 0.01; ***p < 0.001; ****p < 0.0001.

To take a closer look at how SR/hnRNP knockdown impacts the macrophage transcriptome during *Salmonella* infection, we quantified the number of transcripts whose abundance was increased or decreased in the absence of each SR or hnRNP, compared to a SCR control (p<0.05). As visualized in **Figures 3A–E** (SRs) and **4A–E** (hnRNPs), we found that each SR and hnRNP queried can act as either a positive or negative regulator of gene expression To identify the most impacted DEGs, we generated heatmaps that show the top 10 most up and down DEGs (p<0.05) in each *Salmonella*-infected SR/hnRNP knockdown macrophage cell line compared to SCR (**Figures 3A–E** and **4A–E**). We then annotated innate immune-responsive genes by virtue of their being up- or down-regulated in control macrophages in response to *Salmonella* infection (+/- 2.0 fold in SCR SAL vs. SCR UN; bolded genes in heatmaps). These heatmaps show clear hyper- or hypo-induction of many critical innate immune genes in SR/hnRNP knockdown macrophages (**Figures 3A–E** and **4A–E**). We validated the expression of a representative “top 10” DEG by RT-qPCR (**Figures 3A–E** and **4A–E**) using two different knockdown cell lines for each SR/hnRNP (efficiency of each knockdown at the RNA level, and protein level (when antibodies were readily accessible) is shown in **Figures S3A–E** and **S4A–E**).

Several interesting trends emerge from these data. First, we found that knockdown of SR and hnRNPs impacts expression of innate immune genes from distinct transcriptional regulons: NFκB (e.g. *Plau*, *Olr1*, *Csf2*, *Csf3*) and IRF3 (e.g. *Ifit1*, *Ifit3*, *Apol9a/b*, *Mx1*). Second, by creating Venn diagrams to identify common DEGs, we found that the hnRNPs queried share more DEGs than do the SRs (**Figures 3F** and **4F**; 104 vs. 11). This result echoes previous global analyses of hnRNP A1, A2/B1, F, H1, M, and U targets in human 293T cells, which described considerable cooperation between hnRNP family members (35). Third, we discovered that certain genes, particularly those categorized as ISGs (36–38), including the viral restriction factors *Mx1*, *Ifit1*, *Ifit3*, and *Oasl2*, and the cytosolic DNA sensor *Zbp1*, show altered abundance in multiple SR/hnRNP knockdown cell lines. Lastly, we found that loss of either SR proteins (**Figure 3G**) or hnRNPs (**Figure 4G**) is more likely to impact steady state levels of genes that are upregulated (>2-fold) at 4h post-*Salmonella* infection than those that are downregulated (>2-fold).

### SR/hnRNP-Mediated Alternative Splicing Events Are Not Common in DEGs

Data presented so far generally argue against a global up- or down-regulation of pre-mRNA splicing in *Salmonella*-infected macrophages and instead support a model whereby individual SRs and hnRNPs dictate RNA processing decisions for particular transcripts. While SR and hnRNP proteins have been implicated in many steps of gene expression and RNA processing, from chromatin remodeling and transcription to mRNA export and stability (39–42), the main way that SR and hnRNPs shape the steady state transcriptome is by influencing pre-mRNA splicing decisions. To begin to appreciate how SR and hnRNPs mediate specific alternative splicing events in murine macrophages and determine whether these events are changed during *Salmonella* infection, we employed an algorithm to identify and quantify local splicing variations (LSV) called Modeling Alternative Junction Inclusion Quantification (MAJIQ) (26). MAJIQ allows identification, quantification, and visualization of diverse LSVs, including alternative 5′ or 3′ splice site usage, exon skipping, and intron retention across different experimental conditions.

**Figure 5:**
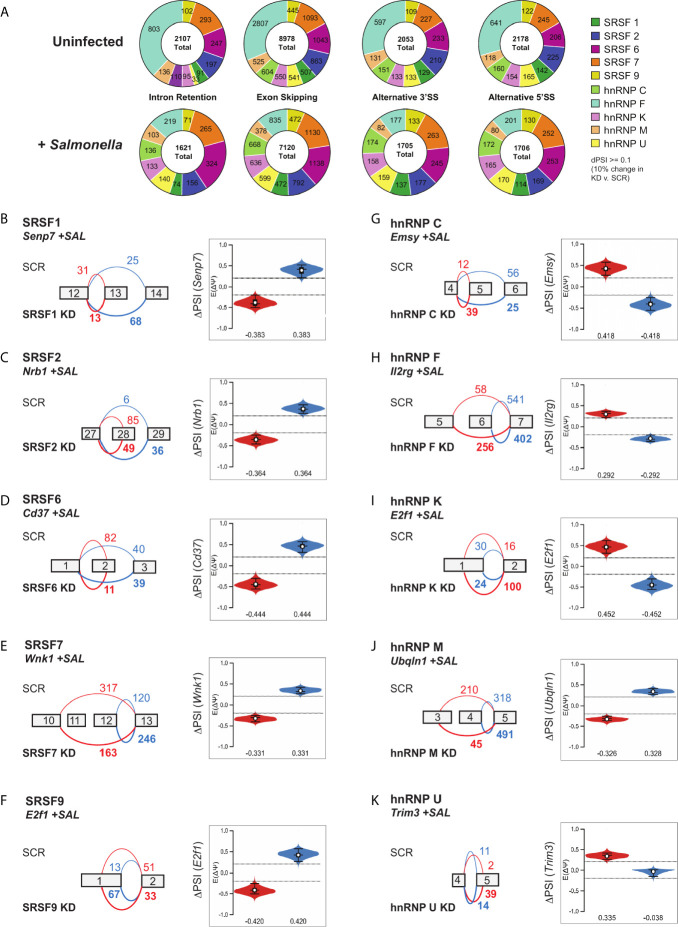
Local splicing variations are abundant in SR/hnRNP knockdown macrophages, but they do not preferentially occur in SR/hnRNP-dependent differentially expressed genes. **(A)** Quantitation of intron retention, exon skipping, alternative 3’ and 5’ splice site events in uninfected and *Salmonella*-infected SR and hnRNP knockdown macrophages (probability [∣delta PSI∣, ≥10%], >95%). PSI is defined as “Percent Spliced In” and indicates the abundance of a particular alternatively spliced isoform. ΔPSI indicates the abundance of an isoform in knockdown vs. SCR macrophage cell lines. **(B)** (left) VOILA output, based on RNA-seq reads, of affected exons in a representative gene (Senp7) in SRSF1 knockdown or SCR RAW 264.7 cells infected with *Salmonella*. (right) Violin plots depicting the ΔPSI of the SRSF1-dependent local splicing variations of *Senp7*. Violin plot colors correspond to the events depicted in the gene schematic on the left. **(C)** As in **(B)** but for SRSF2 and *Nrb1*. **(D)** As in **(B)** but for SRSF6 and *CD37*. **(E)** As in **(B)** but for SRSF7 and *Wnk1.* **(F)** As in **(B)** but for SRSF9 and *E2f1.* **(G)** As in **(B)** but for hnRNP C and *Emsy.* **(H)** As in **(B)** but for hnRNP F and *Il2rg.* **(I)** As in **(B)** but for hnRNP K and *E2f1.* **(J)** As in **(B)** but for hnRNP M and *Unqln1.* **(K)** As in **(B)** but for hnRNP U and *Trim3.*

Using MAJIQ, we quantified LSVs that were significantly changed in SR/hnRNP knockdown cells in both uninfected and *Salmonella*-infected conditions (4h post-infection), generating a large dataset of SR/hnRNP-dependent alternative splicing changes (probability [∣delta PSI∣, ≥10%] >95%) (**Supplementary Table 1**). We observed all types of alternative splicing changes, with the majority of changes categorized as exon skipping events (>7000 total exon skipping events in UN or +SAL vs. >1600 intron retention, alternative 5’ and 3’SS events in UN or +SAL), consistent with the canonical roles of SR and hnRNPs in enhancing or repressing exon inclusion (**Figure 5A**) (43–45). There were no dramatic differences between the overall number of LSVs between different SR/hnRNPs nor major differences in the number of LSVs in uninfected compared to *Salmonella*-infected macrophages (**Figure 5A**). HnRNP F knockdown macrophages stood out as a notable exception, whereby there were about one-quarter as many LSVs identified in *Salmonella*-infected macrophages vs. uninfected (**Figure 5A**).

Recent work from the Baltimore lab showed that innate immune transcripts are frequently regulated by alternative splicing events whereby poison exons introduce premature stop codons that target transcripts to nonsense mediated decay (22). To determine whether alternative splicing events could be contributing to differential gene expression in SR/hnRNP knockdown macrophages, we compared our lists of DEGs and genes with DIU in both conditions and visualized the overlap by generating Venn diagrams. Surprisingly, we observed that only a small fraction of SR/hnRNP-dependent DEGs were also subject to DIU (i.e. few transcripts were impacted at levels of both steady state abundance and alternative splicing) (**Figure S5A**). These trends were the same in uninfected and *Salmonella*-infected SR/hnRNP knockdown macrophages (**Figures S5A, B**). In line with this finding, we observed little to no enrichment for SR/hnRNP-dependent alternative splicing events in genes related to innate immune pathways *via* IPA. In fact, no pathway was enriched for genes with DIU more than -log(p-value) = 5 (**Supplementary Table 1**), suggesting that SR/hnRNP-dependent alternative splicing changes in macrophages do not generally occur in functionally related genes from any particular pathway. Other studies have also found a lack of overlap between steady state transcript level changes and alternative splicing events in *Salmonella*-infected human monocytes (21) as well as in influenza-infected A549 cells (46).

To gain additional insight into how SRs and hnRNPs influence macrophage biology during infection, we next looked at the most significant DIUs in each *Salmonella-*infected knockdown cell line using a more stringent isoform expression cut-off (probability [∣delta PSI∣, ≥20%] >95%). As supported by our pathway analysis, these genes fall into a variety of functional categories, including protein modification, intracellular trafficking, chromatin remodeling and transcription, and chromosome biology (**Figure S5D**). Notably, there are no obvious candidates for genes likely to globally alter the innate immune transcriptome, save for *Ikbke* (inhibitor of nuclear factor kappa-B kinase subunit epsilon), which is subject to intron retention in hnRNP F knockdown cells. A representative DIU in an innate immune-related gene (>20% delta PSI) is shown for each SR/hnRNP knockdown cell line (**Figures 5B–K**). Several of these splicing variations influence the protein coding capacity of their targets, with changes to the balance of exon skipping events in SRSF knockdowns (*Senp7* in SRSF1, **Figure 5B**; *Nrb1* in SRSF2, **Figure 5C**; *Cd37* in SRSF6, **Figure 5D**; and *Wnk1* in SRSF7, **Figure 5E**) and hnRNP knockdowns (*Emsy* in hnRNP C, **Figure 5G**; *Il2rg* in hnRNP F, **Figure 5H**; and *Ubqln1* in hnRNP M, **Figure 5J**). Interestingly, while most DIUs were detected in both uninfected and *Salmonella*-infected SR/hnRNP knockdown macrophages, changes in SRSF2 dependent-changes to *Nrb1*, SRSF9-dependent changes to *E2f1*, hnRNP C-dependent changes to *Emsy*, hnRNP K-dependent changes to *E2f1* hnRNP K, and hnRNP U-dependent changes to *Trim3* were only found in +SAL samples (**Figures 5C, F, G, I, K**) (**Supplementary Table 1**). Together, these data suggest that while SR/hnRNPs do not control macrophage transcript abundance *via* changes to alternative splicing, these factors can mediate distinct alternative splicing events in uninfected vs. *Salmonella*-infected macrophages.

### A Gene’s Induction Level, Length, and Number of Exons/Introns Do Not Correlate With a Transcript’s Reliance on SR/hnRNPs for Proper Induction During *Salmonella* Infection

**Figure 6:**
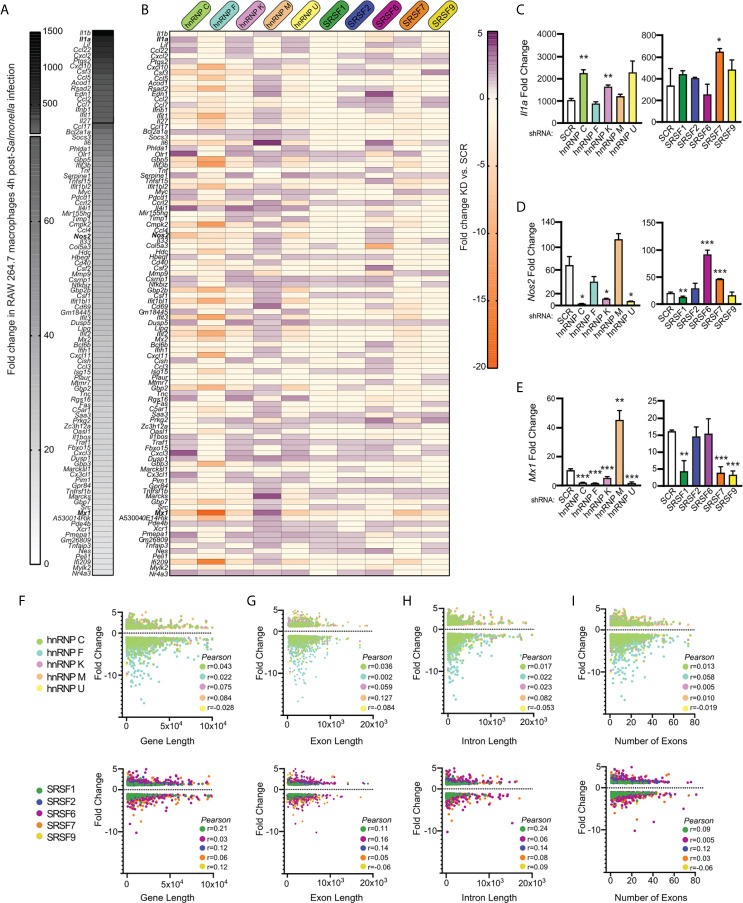
Level of induction upon *Salmonella* infection, gene length, and number of introns/exons do not positively correlate with a gene’s reliance on SR/hnRNPs to maintain proper expression levels. **(A)** Heatmap of the top 100 genes induced at 4h post-*Salmonella* infection in SCR control RAW 264.7 macrophages. Data shown as fold change in SCR cells + *Salmonella* vs. SCR cells uninfected. **(B)** Heatmap of up- or down-regulation (fold change) of each induced gene conferred by hnRNP or SRSF knockdown. Purple genes are upregulated in SR/hnRNP knockdowns relative to SCR; Orange genes are downregulated in SR/hnRNP knockdowns. **(C)** RT-qPCR of *Il1a* abundance relative to *Actb* in *Salmonella*-infected SRSF and hnRNP knockdown macrophages (shown as fold change relative to uninfected for each cell line). **(D)** As in **(C)** but for *Nos2*. **(E)** As in **(C)** but for *Mx1*. **(F)** Scatter plot depicting correlation of the fold change of each DEG vs. coding sequence (CDS) length in *Salmonella*-infected hnRNP (top) and SRSF (bottom) knockdown macrophages. **(G)** as in **(F)** but comparison of DEG fold change vs. exon length. **(H)** As in **(F)** but comparison of DEG fold change vs. intron length. **(I)** As in **(F)** but comparison of DEG fold change vs. number of exons in a DEG. Genes included in analysis were differentially expressed (p < 0.05) in each knockdown compared to SCR controls. Y-axes were made all the same to facilitate comparison between multiple knockdown cell lines. For all RT-qPCRs values are the mean of 2 or 3 biological replicates and error bars indicate standard deviation. *p < 0.05; **p < 0.01; ***p < 0.001.

Having observed a lack of correlation between DEGs and DIU in each splicing factor knockdown cell line, we wanted to see if we could identify anything common to SR/hnRNP-sensitive innate immune genes. We hypothesized that genes whose expression is the most upregulated in response to *Salmonella* infection could be more sensitive to loss of SR/hnRNPs, perhaps *via* a need to sequester rate-limiting spliceosome components. To address this possibility, we first ranked all genes induced in SCR control macrophages at 4h post-*Salmonella* infection (**Figure 6A**). We observed dramatic upregulation of hundreds of macrophage genes at this early time point, with some inflammatory mediators like *Il1a* and *Il1b* upregulated approximately 1500-fold. We then generated another heatmap to visualize how the expression of each of these top 100 *Salmonella*-induced genes was impacted by SR/hnRNP knockdown. We observed no clear correlation between level of induction/expression level and whether or not a transcript was differentially regulated by loss of an SR/hnRNP. This is clearly evidenced by the heatmap itself, whereby DEGs induced 1000-fold and DEGs induced 5-fold in control cells were similarly impacted, both in terms of the number of SR/hnRNPs they were affected by and the magnitude of their expression change (**Figure 6B**; top vs. bottom genes). We can also see this outcome in RT-qPCR experiments, in which we measured how loss of SR/hnRNPs impacted expression of *Il1a* (500-1000 average fold-change in SCR controls; **Figure 6C**), *Nos2* (Nitric oxide synthase) (20-60 average fold-change; **Figure 6D**), and *Mx1* (MX Dynamin Like GTPase 1) (10-15 average fold-change; **Figure 6E**). Each of these representative innate immune genes responds to loss of particular SRs and hnRNPs in completely different ways. For example, loss of hnRNP M causes hyper-induction of *Mx1*, but does not affect *Il1a* levels. Loss of hnRNP C causes hyper-induction of *Il1a* but does not impact *Nos2* or *Mx1* abundance. Using the computational prediction software RBPmap (27), we successfully identified one or more binding sites for each of the SR/hnRNPs that impacted *Il1a*, *Nos2*, and/or *Mx1* expression (**Figures S6A–C**). While this analysis is merely correlative, it does begin to support a model whereby exonic and intronic splicing enhancers/silencers are enriched in innate immune transcripts that rely on particular SR/hnRNPs for proper expression levels.

To examine if other attributes of a gene influenced whether its expression was altered by loss of an SR/hnRNP, we conducted Pearson’s correlation tests to determine the relationship between differential expression (p<0.05) and gene length (**Figure 6F**), exon length (total exonic sequence, or sum of all exon nucleotides) (**Figure 6G**), intron length (total intronic sequence, or sum of all intron nucleotides) (**Figure 6H**), and number of exons (**Figure 6I** and **Supplementary Table 1**). We observed little to no correlation between any of these gene attributes and the degree to which a gene’s expression was altered in the hnRNP and SR knockdown cell lines, with all tests generating Pearson correlation coefficients close to zero. Thus, it is likely that additional features, for example the presence or absence of specific binding sites/consensus sequences or misregulation of an upstream transcription or chromatin factor, dictate whether an innate immune transcript is sensitive to loss of a particular SR/hnRNP.

### SR/hnRNP Knockdown Leads to Hyper-Induction of Early-Induced Innate Immune Genes

**Figure 7:**
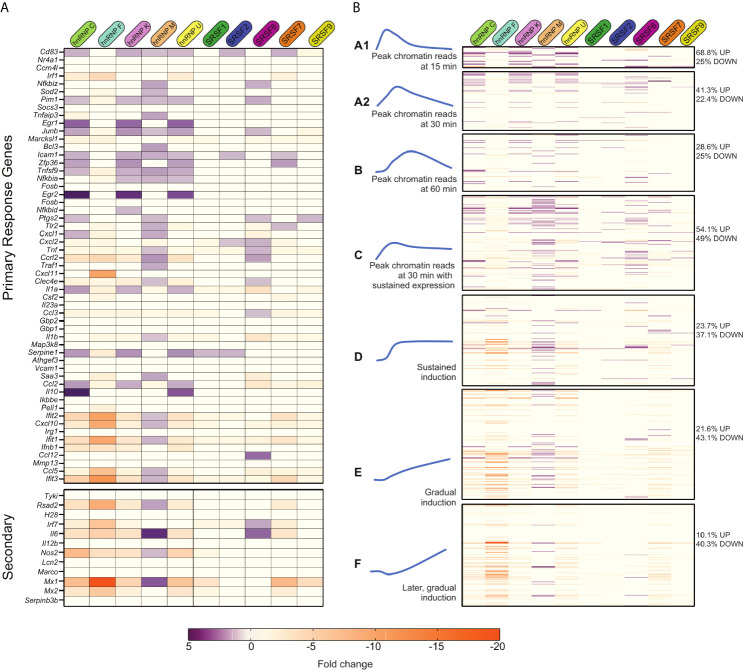
Many primary response and early induced innate immune genes are repressed by SR/hnRNPs. **(A)** Fold change of SR/hnRNP DEGs in *Salmonella*-infected RAW 264.7 macrophages compared to SCR controls for genes categorized as primary (top) and secondary (bottom) response genes according to 10. 35/53 primary response genes are upregulated by loss of one or more SR/hnRNPs at 4h post-*Salmonella* infection. **(B)** Fold change of SR/hnRNP DEGs in *Salmonella*-infected RAW 264.7 macrophages compared to SCR controls with Lipid A-induced genes categorized on basis of induction kinetics as defined by 6. Percentages on right indicate the number of genes in each category that are differentially expressed (up- or down-regulated) by loss of one or more SR/hnRNP. (left) Blue curves are schematic representations of the induction kinetics of each Group (A1, A2, B, C, D, E, and F) over a 120-minute time course following Lipid A treatment. Adapted from 6.

With no apparent correlation between various gene architecture attributes and SR/hnRNP reliance, we looked to see if we could correlate an innate immune gene’s reliance on SR/hnRNPs for proper induction with the dynamics of its transcriptional activation as previously described by other studies (6, 7, 10). One commonly used categorization of innate immune genes is into primary and secondary response genes. Of the 53 LPS-driven primary response genes annotated by (10), 35 of them were more abundant in one or more SR/hnRNP knockdown cell line (**Figure 7A**, top heatmap; **Supplementary Table 1**). This suggests a role for factors like hnRNP C, K, M, U and SR6 in repressing the expression of primary response genes at the 4h time point we interrogated. A repressive role for these same factors was less evident for secondary response genes, which were mostly downregulated in the absence of the SR/hnRNPs (save for hnRNP M and SRSF6) (**Figure 7A**, bottom heatmap; **Supplementary Table 1**). This analysis begins to suggest that primary response genes may be particularly reliant on pre-mRNA splicing to control the proper magnitude of their induction than are secondary response genes, which rely on multiple additional layers of regulation.

We next looked at our data in a different way, leveraging macrophage gene categories as defined by (6). This study divided RefSeq genes exceeding 400bp in length into 12 clusters based on their pattern of transcript levels in three cellular compartments (chromatin, nucleoplasm, cytoplasm) in primary macrophages over a time-course of Lipid A treatment (**Figure S7A** and **Supplementary Table 1**). Resorting our own data into these groups, we found that the vast majority of genes we identified as SR/hnRNP-sensitive in *Salmonella*-infected RAW 264.7 macrophages fell into Groups 1-3 (**Figure S7A** and **Supplementary Table 1**), which are composed mainly of Lipid A-induced genes. This supports our hypothesis that SR/hnRNPs play a specific role in controlling macrophage genes that are activated upon pathogen sensing.

To gain additional insight into how different categories of induced genes respond to SR/hnRNP knockdown, we re-sorted our data again, this time using a more detailed grouping of macrophage induced genes from Bhatt et al. These groups of genes (A1-F; **Figure 7B** and **Supplementary Table 1**) were defined on basis of when chromatin-associated RNA-seq reads for each gene reach peak levels following Lipid A treatment: Group A1 peaks at 15 min, A2 at 30 min, B at 60 min, C and D levels peaked around 30 min and then were sustained through 2h, and Groups E and F steadily increased over the 2h time-course (represented schematically in **Figure 7B**). One interesting trend that emerged from this data is that the majority of SR/hnRNP-sensitive genes in Groups A1-C were hyper-induced (68.8% of Group A1 genes were upregulated in one or more knockdown macrophage cell line vs. SCR; 41.3% in Group A2 and 54.1% in Group C). This suggests that LPS-activated genes expressed *via* a transcriptional “burst” are uniquely sensitive to repression from SR/hnRNPs, in particular hnRNP C, hnRNP K, hnRNP M, hnRNP U, SRSF2, 6, and 7. We also found that genes in Group C were disproportionately impacted by loss of hnRNP M, with nearly half of all genes in the category (40/98) upregulated in hnRNP M knockdown macrophages at 4h post-*Salmonella* infection, including the chemokine receptor *Ccrl2*, the regulator of NFκB *Nfkbiz*, and the repressor of JAK/STAT signaling *Socs3*.

### hnRNP U and hnRNP K Play a Role in the Control of Intracellular Viral and Bacterial Replication

Lastly, we wanted to test whether we could use DEG and/or DIU profiles of SR/hnRNP knockdown macrophages to predict if a particular knockdown cell line would be better or worse at restricting pathogen replication. We began by applying a simple hierarchical clustering algorithm to calculate similarities in DEG profiles between knockdowns (Cluster 3.0). We found significant similarity between genes affected by loss of hnRNP K and hnRNP U (and to a lesser extent, hnRNP C) in uninfected cells (correlation between K/U: 0.78; correlation between C/K/U: 0.69). Previous studies have shown that hnRNP K binds strongly to poly C stretches of RNA (47, 48) and hnRNP U preferentially binds CUGUGGAU and UGUAUUG motifs (35). At the amino acid level, hnRNP K and U proteins are only 31% similar in mice (EMBOSS Stretcher Pairwise Sequence Alignment). While their consensus binding motifs argue against their recognizing overlapping sequences, there is evidence from high-throughput studies in humans and mice that hnRNP K and U proteins immunopurify (49) and cofractionate (50, 51) together.

**Figure 8:**
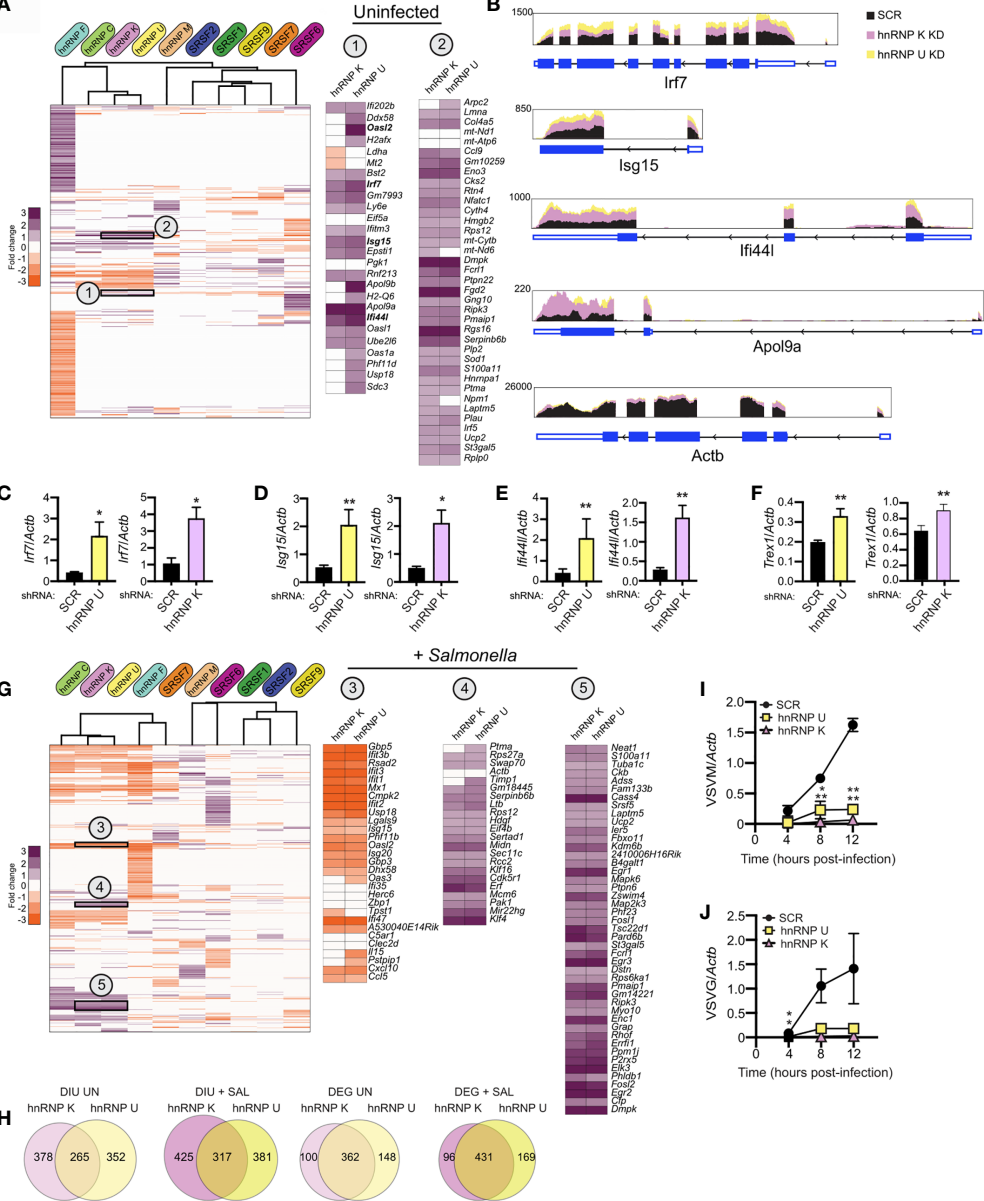
Loss of hnRNP K and hnRNP U affects similar DEGs and DIU and impacts the ability of RAW 264.7 macrophages to control viral replication. **(A)** Hierarchical clustering of up- and down-regulated DEGs in uninfected macrophages (SR/hnRNP knockdown vs. SCR). Zoom-ins of clusters of interest (1 and 2) are shown to the right. Correlation of Cluster 1 is 0.84; Cluster 2 is 0.76. To allow for better visualization of DEGs, the scale is set at -3 to +3. **(B)** IGV tracks showing RNA-seq reads for *Irf7*, *Isg15, Ifi44l*, and *Apol9a* in hnRNP K and hnRNP U compared to SCR control reads. *Actb* is included as a representative unaffected control gene. **(C)** RT-qPCR validation of *Irf7* basal expression in hnRNP U and K knockdown cell lines vs. SCR controls. Data is displayed as *Irf7/Actb*. **(D)** As in **(C)** but for *Isg15*. **(E)** as in **(C)** but for *Ifi44l*. **(F)** as in **(C)** but for *Trex1*. **(G)** As in **(A)** but for *Salmonella*-infected macrophages. Correlation of Cluster 3 is 0.75; Cluster 4 is 0.78; Cluster 5 is 0.72. **(H)** Overlap of DIUs and DEGs in uninfected and *Salmonella*-infected hnRNP K and U knockdown macrophages. *(I)* Vesicular Stomatitis Virus (VSV) replication (as measured by RT-qPCR of the VSVM gene relative to *Actb*) in hnRNP K and hnRNP U knockdown macrophages compared to SCR controls (VSV MOI = 1) at 4, 8, and 12h post-infection. **(J)** as in **(I)** but for VSVG. For all RT-qPCRs, values are the mean of 3 biological replicates and error bars indicate standard deviation. *p < 0.05; **p < 0.01.

To determine if hnRNP K and U knockdown RAW 264.7 cell lines may be phenotypically similar, we identified several clusters of up- and down-regulated genes common to both cell lines. Two clusters of upregulated genes are highlighted in **Figure 8A**. Interestingly, Cluster 1 contains mostly ISGs (*Ifi202b*, *Bst2*, *Irf7*, *Ifitm3*, *Isg15*, *Ifi44l*, *Oasl1*) while Cluster 2 is enriched for a diverse group of kinases (*Dmpk*, *Ripk3*), regulators of GTPase activity (*Gng10*, *Rgs16*, *Fgd2*), and mitochondrial related factors (*Pmaip1, Ucp2*). Differential expression of ISGs in uninfected hnRNP K and U knockdown cell lines is notable because it suggests that loss of these factors somehow activates macrophages to upregulate type I interferon stimulated genes (see model in **Figure 1B**). This basal ISG phenotype can also be appreciated by looking at RNA-seq reads *via* the Integrated Genome Viewer (Broad), whereby *Isg15*, *Ifi44i*, and *Apol9a* are expressed at 2-3-fold higher than SCR controls in both hnRNP K and hnRNP U knockdown uninfected macrophages, but *Actb* showed no difference in expression (**Figure 8B**). We confirmed this high basal ISG phenotype *via* RT-qPCR in both hnRNP K and U knockdown cell lines for several representative ISGs: *Irf7* (**Figure 8C**), *Isg15* (**Figure 8D**), *Ifi44l* (**Figure 8E**), and *Trex1* (**Figure 8F**).

While the profiles of SR/hnRNP-sensitive genes change dramatically upon *Salmonella* infection, we still observe significant correlation between genes impacted by loss of hnRNP K and hnRNP U (**Figure 8G**) (correlation between K/U: 0.79; correlation between C/K/U: 0.76). Interestingly, upon *Salmonella* infection, several of the Cluster 1 ISGs actually became less abundant relative to SCR controls (**Figure 8G**; *Oasl2*, *Ddx58*, *Isg15*, *Usp18*; +SAL Cluster 3), highlighting dysregulation of specific type I interferon genes in the absence of hnRNP K and U. Cluster 2 DEGs, on the other hand, were more abundant in both uninfected and *Salmonella*-infected hnRNP K and U knockdown macrophages (**Figure 8G**; +SAL Clusters 4 and 5). Overlap between hnRNP K and U DEGs and DIUs is illustrated by Venn diagrams that show that one-third of total DEGs are shared and three-fourths of DIUs (**Figure 8H**).

A major role for interferon stimulated genes is controlling viral replication through a variety of restriction mechanisms [e.g. limit viral entry, inhibit replication of the viral genome, interfere with host cell translation, etc. (36)]. Thus, we asked whether viral replication was impacted at early infection time points in hnRNP K and U knockdown cell lines compared to SCR controls. We infected SCR, hnRNP K and hnRNP U knockdown RAW 264.7 macrophages with vesicular stomatitis virus (VSV) at a MOI of 1 and measured viral replication over a 12h time course by quantifying expression of two viral genes, VSV-G and VSV-M by RT-qPCR. VSV is a single-stranded, enveloped RNA virus that can replicate and elicit robust gene expression changes in RAW 264.7 cells (52). Remarkably, we observed almost no replication of VSV in either hnRNP knockdown cell line at any time point (**Figures 8I, J**). A similar hyper-restriction phenotype was recently reported for hnRNP K knockdown A549 cell lines infected with influenza virus by (46). Thompson et al. attribute this phenotype to hnRNP K-dependent alternative splicing of a number of genes required for viral replication. Notably, we detect DIUs for several of the same genes (Setd5, Arhgap12, Gpbp1, and Eri2) (**Supplementary Table 1**), suggesting that hnRNP K’s role in viral infection is conserved between mice and humans and may, in part, be mediated by the same alternative splicing events.

**Figure 9:**
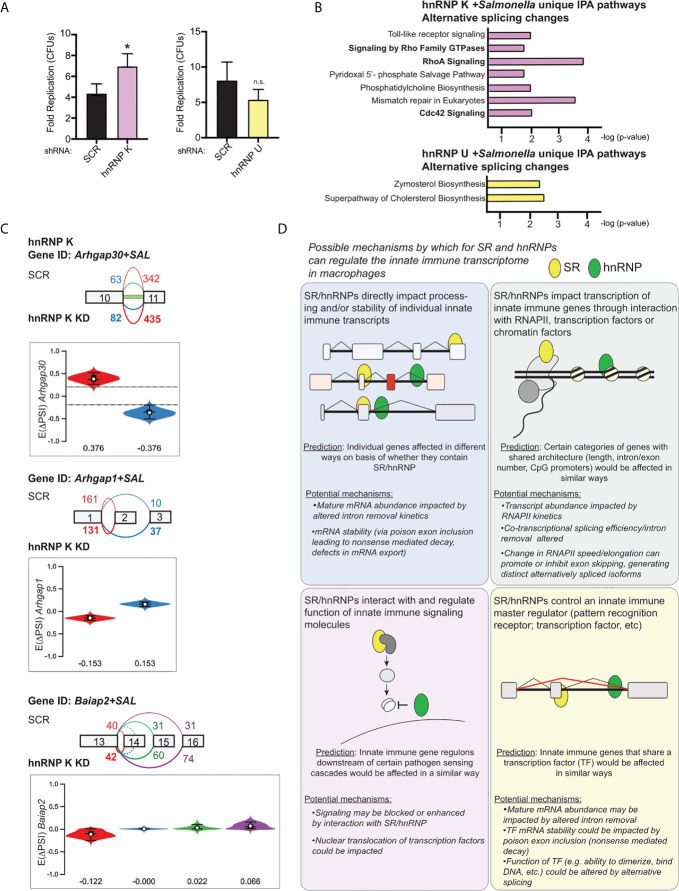
hnRNP K regulates *Salmonella* replication and controls alternative splicing of genes involved in RhoA/Rac1-mediated reorganization of the actin cytoskeleton. **(A)** Colony forming units represented as fold replication (20h time point relative to 2h time point) in hnRNP K and hnRNP U knockdown RAW 264.7 macrophages infected with *Salmonella* Typhimurium (MOI = 10) **(B)** Ingenuity Pathway Analysis of hnRNP K (top) and hnRNP U (bottom) knockdown macrophages at 4h post-*Salmonella* infection. Pathways shown are unique to hnRNP K or hnRNP U cells and enrichment is shown as -log(p-value). **(C)** Local splicing variations identified in three genes related to Cdc42-mediated reorganization of the actin cytoskeleton: Arhgap30, Arhgap1, and Baiap2. Color of splicing events in the gene schematic corresponds to the colors in the violin plots (showing EΔPSI). **(D)** Model of the potential mechanisms through which SR and hnRNP family members could impact innate immune gene expression in macrophages. For all RT-qPCRs values are the mean of 2 or 3 biological replicates and error bars indicate standard deviation.*p < 0.05; n.s. is not statistically significant.

Lastly, we wanted to determine whether loss of hnRNP K or U impacted the outcome of *Salmonella* infection. We infected SCR, hnRNP K knockdown, and hnRNP U knockdown RAW 264.7 cell lines with an overnight culture of *Salmonella* (53) at an MOI of 10 and measured colony forming units (CFUs) at 2h (a measure of internalization) and 20h (a measure of intracellular replication) post-infection. We observed an almost two-fold increase in *Salmonella* replication in hnRNP K knockdown macrophages relative to SCR controls, but no apparent difference attributable to loss of hnRNP U (**Figure 9A**). Leveraging our transcriptomics and alternative splicing analysis, we looked to see if we could identify changes that were unique to hnRNP K. We observed that hnRNP K knockdown in RAW 264.7 macrophages preferentially impacted alternative splicing events of genes involved in RhoA and Cdc42 signaling (**Figure 9B**, top). These top enriched categories were very different from those in hnRNP U, which showed enrichment for DIUs in genes involved in cholesterol biosynthesis (**Figure 9B**, bottom). RhoA and Cdc42 are Rho GTPases that co-ordinate cytoskeletal dynamics (54). We detected differential isoform usage for several transcripts involved in these pathways including *Arhgap30*, *Arhgap1*, and *Baiap2* (**Figure 9C**). In fact, the intron retention event in *Arhgap30* is the one of the most abundant DIUs in hnRNP K knockdown macrophages (E(ΔPSI) 0.376) (**Supplementary Table 1**). This DIU is predicted to decrease the relative abundance of protein coding-competent *Arhgap30* mRNA in the cell, while events in *Arhgap1* and *Baiap2* are predicted to change the amino acid sequence of the protein isoform. Our finding that *Salmonella* can replicate more efficiently in the presence of these alternative splicing changes is consistent with work showing that actin polymerization is critical for stabilization of the *Salmonella*-containing vacuole and replication of intravacuolar *Salmonella* in cells like macrophages (55, 56). These data suggest that hnRNP K may impact actin dynamics through alternative splicing of *Arhgap30*, *Arhgap1*, and *Baiap2*. Furthermore, our results argue that individual splicing factors can contribute to innate immune and infection outcomes in unique ways and demonstrate that together, transcriptomics and alternative splicing analysis has the potential to identify host factors that regulate the host-pathogen interface.

## Discussion

Our study illustrates the diverse effects the SR/hnRNP family splicing factors have on the macrophage transcriptome both in uninfected macrophages and in cells infected with the gram-negative bacterial pathogen *Salmonella* Typhimurium. While implicating SR and hnRNPs in regulating gene expression is not remarkable, the degree to which each of the splicing factors queried impacts distinct gene regulons in each of these conditions is unexpected. Overall, we found that over 70% of the genes induced as part of the macrophage innate immune response (>2-fold at 4h post-infection) are hyper- or hypo-induced in the absence of one or more of the SR/hnRNPs we investigated. This work highlights a critical role for splicing regulatory proteins in controlling the magnitude of innate immune gene induction and calls for a rethinking of how the innate immune response is post-transcriptionally regulated.

One critical lingering question raised by these studies relates to the mechanism(s) through which genes are up or down regulated in SR/hnRNP knockdown RAW 264.7 macrophages. While our analysis did identify DIU in transcripts encoding several transcription factors in the IRF, STAT, and NFκB families (*Irf1*, *Irf5*, *Irf7*, *Irf9*, and *Stat1*), our data does not generally support a model whereby loss of a particular SR/hnRNP results in mis-splicing or functional alteration of a master regulator of a shared transcriptional regulon. Likewise, although individual cases likely exist, our data does not support a role for SR/hnRNPs in globally regulating innate immune transcript abundance *via* differential inclusion of poison exons (as DIU was not enriched in DEGs). Indeed, the most overlap between DIU and DEG we observed was for hnRNP F and even that was only 10% (**Figure S5**). Thus, the question remains: how do individual SR/hnRNPs activate and/or repress induction of innate immune genes? Several mechanisms are likely and are by no means mutually exclusive (**Figure 9D**). The first possible mechanism driving at least some of these changes is direct binding of SR/hnRNPs to target pre-mRNAs to promote or inhibit constitutive intron removal. Second, certain SRs and hnRNPs could contribute to transcriptional changes by interacting with RNA polymerase and/or other factors at the chromatin level. Indeed, to promote co-transcriptional splicing, some SR proteins interact with the CTD of RNA polymerase II (57–59) and some SR proteins have been shown to interact with histones (60). Third, SR/hnRNPs may alter innate immune gene expression by interacting with components of innate immune signaling cascades themselves, such as pathogen recognition receptors or downstream kinases/transcription factors. Such a mechanism was recently described for hnRNPA2B1, which controls initiation of the type I IFN response in part *via* interactions with the kinase TBK1 and its substrate IRF3 (61). Lastly, although our data does not provide strong support for such a model, it is possible that loss of SR/hnRNPs alters the abundance or function of innate immune transcription factors themselves at either the RNA or protein level. Implicating one or more of these mechanisms will require detailed follow-up analysis on individual SR/hnRNPs. Identifying the direct RNA and protein binding targets of these splicing factors in uninfected and *Salmonella*-infected macrophages will certainly help inform on these mechanisms, as will defining the subcellular localization of these RNA-binding proteins in the two conditions.

Previous work from our lab found hnRNP M knockdown leads to hyper-induction of a number of innate immune transcripts following inflammatory triggers, which suggests that slowing/inhibiting pre-mRNA maturation may be involved. Specifically, our earlier work found that overexpression of hnRNP M promotes accumulation of intron-containing *Il6* pre-mRNAs, while loss of hnRNP M increases removal of *Il6* intron 3 (31). From these findings we concluded that by repressing constitutive intron removal in *Il6*, hnRNP M slows early or spurious transcriptional activation of this pro-inflammatory cytokine. It is possible that other SR and hnRNPs work in the same way, by contributing to specific constitutive intron removal events that can fine-tune the kinetics of transcript maturation and influence steady state RNA levels. Such a model would predict increased levels of reads from particular introns in DEGs. Although we do not see evidence for this in our RNA-seq data, this is not particularly surprising given the low abundance of intron-containing pre-mRNAs relative to mRNAs. Indeed, an important caveat of these studies is that they were carried out at a single time point following *Salmonella* infection. While we chose this time point to maximize mRNA transcript accumulation, we may have inadvertently minimized our ability to detect transient accumulation of unprocessed transcript intermediates. Kinetic transcriptome analysis from the Black and Smale labs demonstrates that for most transcripts, pre-mRNA splicing of innate immune transcripts occurs co-transcriptionally but accumulation of nascent pre-mRNA at the chromatin level is generally not evident at time points following 15-30 minutes, except in select cases with especially long transcripts on which splicing catalysis is delayed (6, 7). Thus, it is possible that for many of our transcripts of interest, the most important contribution of SR/hnRNP proteins to constitutive and/or alternative splicing occurs during that early transcriptional burst, minutes after pathogen sensing. Thus, future attempts to elucidate the complexities of post-transcriptional control of inflammatory gene induction will want to broaden their scope to include additional early time points following macrophage activation.

At the onset of this study, we hypothesized that because SRSF1, 2, 6, 7, 9 and hnRNP C, F, K, M, and U have been shown to be differentially phosphorylated during bacterial and fungal infection of macrophages (23–25), they would impact distinct gene regulons in uninfected vs. S*almonella*-infected cell lines. Several studies have linked environmental changes to post-translational modification (PTM) of splicing factors. For example, dephosphorylation of SRSF10 by the phosphatase PP1 represses splicing and limits gene expression in HeLa cells following heat shock (18, 20, 62). Additionally, arginine methylation of hnRNPA1/B2 triggers its export to the cytoplasm where it activates TBK1/IRF3 signaling following infection with a DNA virus (61). Recent work from the Lynch lab showed that hnRNP K is redistributed in the nucleus during influenza infection, becoming enriched in nuclear speckles (46). Indeed, subcellular redistribution is a common trait of SR/hnRNPs during infection (63, 64) and many viruses themselves require RNA binding proteins for the maintenance and processing of their genomes. Future studies designed to investigate how pathogen sensing influences post-translational modification of SR and hnRNPs will provide important insights into how splicing factors are functionalized during the macrophage innate immune response as well as in response to cellular stresses in general.

## Data Availability Statement

The datasets presented in this study can be found in online repositories. The name of the repository and accession number can be found below: National Center for Biotechnology Information (NCBI) Gene Expression Omnibus (GEO), https://www.ncbi.nlm.nih.gov/geo/, GSE171418.

## Author Contributions

KW and AW generated the knockdown cell lines, carried out *Salmonella* infections and performed RNA sequencing. KW performed bioinformatics analysis. AW, HS, AC, TF, and KC performed experimental validations. KV, KP, and RW contributed to data visualization. KP, RW, AW and HS prepared and edited the figures and manuscript. All authors contributed to the article and approved the submitted version.

## Funding

This work was funded by the National Institutes of Health/NIGMS, R35GM133720 (KLP) and National Institutes of Health/NIAID R01AI125512 (ROW).

## Conflict of Interest

The authors declare that the research was conducted in the absence of any commercial or financial relationships that could be construed as a potential conflict of interest.
