# Supplementary material for: Global Transcriptomics Uncovers Distinct Contributions From Splicing Regulatory Proteins to the Macrophage Innate Immune Response
Source: Front Immunol. 2021 Jul 9;12:656885. doi: 10.3389/fimmu.2021.656885 (PMC8299563; doi:10.3389/fimmu.2021.656885)
Supplement: Supplementary file 1 [file DataSheet_1.pdf]

A

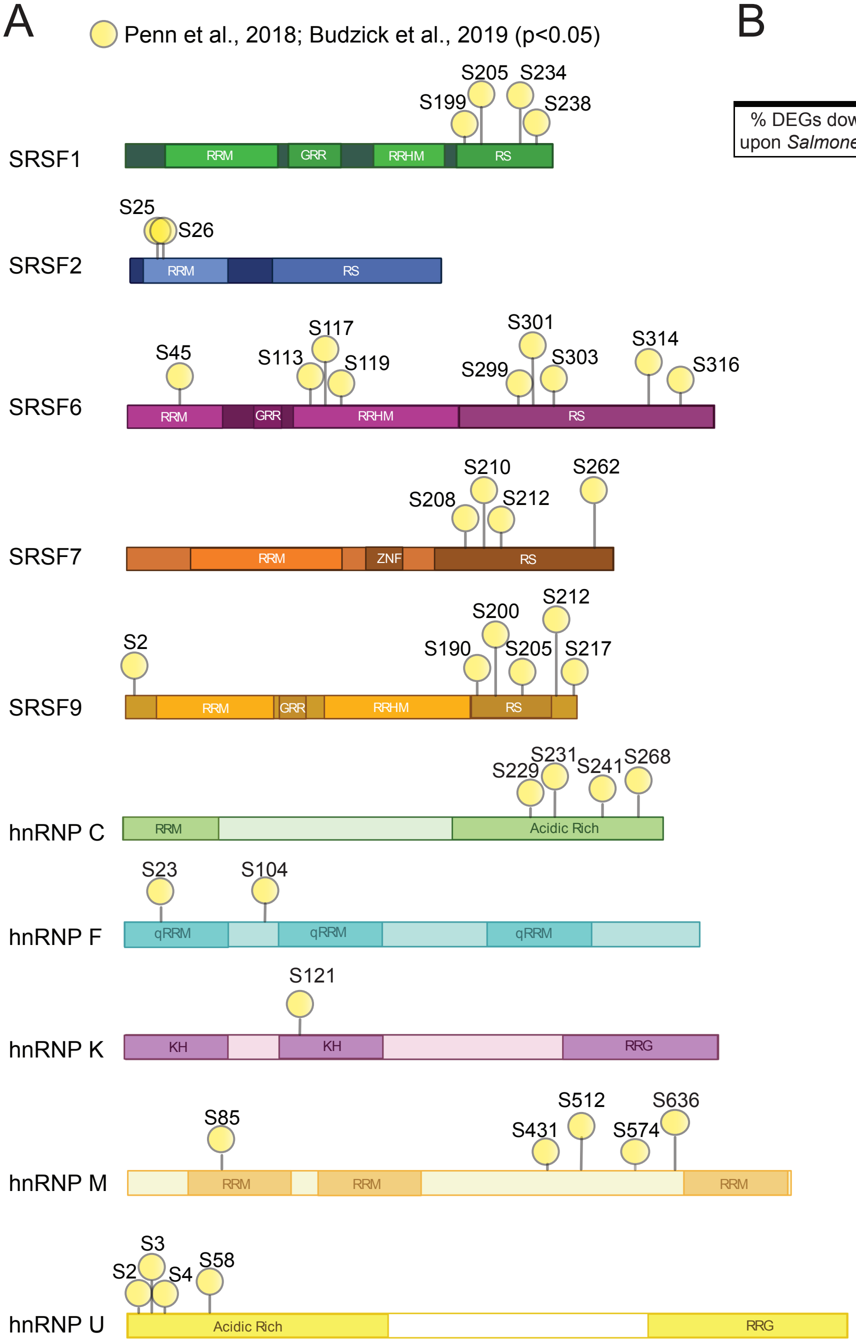

RRM = RNA Recognition Motif
KH = K Homology Domain
qRRM = Quasi RMM
GRR = Glycine Rich Region

RS = Arginine-Serine Rich
RRG = Arginine-Glycine Repeat Region
RRHM = RRM Homology
ZNF = Zinc-Binding Domain

B

	SRSF1	SRSF2	SRSF6	SRSF7	SRSF9	hnRNP C	hnRNP F	hnRNP K	hnRNP M	hnRNP U
% DEGs downregulated upon *Salmonella*-infection	1.3	3.5	1.8	3.2	2.8	4.3	4.7	5.8	4.6	6.3

Figure S1: (A) Schematic representation of sites of differential phosphorylation in *Mycobacterium tuberculosis*-infected macrophages as reported by Penn et al., 2019 and Budzik et al., 2020 for SRSF and hnRNPs queried. Abbreviations for different protein domains are defined below gene diagrams. (B) Percentage of differentially expressed genes (DEGs) in each SR/hnRNP knockdown cell line that are downregulated >2.0-fold in response to *Salmonella* infection in control macrophages.

Figure S1

A

Ingenuity Canonical Pathways	-log(p-value)
Senescence Pathway	5.46
Cell Cycle: G1/S Checkpoint Regulation	5.21
Chronic Myeloid Leukemia Signaling	4.67
Role of BRCA1 in DNA Damage Response	4.64
Cyclins and Cell Cycle Regulation	4.6
Pancreatic Adenocarcinoma Signaling	3.68
Molecular Mechanisms of Cancer	3.51
Colorectal Cancer Metastasis Signaling	3.41
Estrogen-mediated S-phase Entry	3.28

Figure S2: (A) Ingenuity pathway analysis (-log (p-value)) of genes downregulated in *Salmonella* vs. uninfected SCR macrophages (fold change of -2 or more).

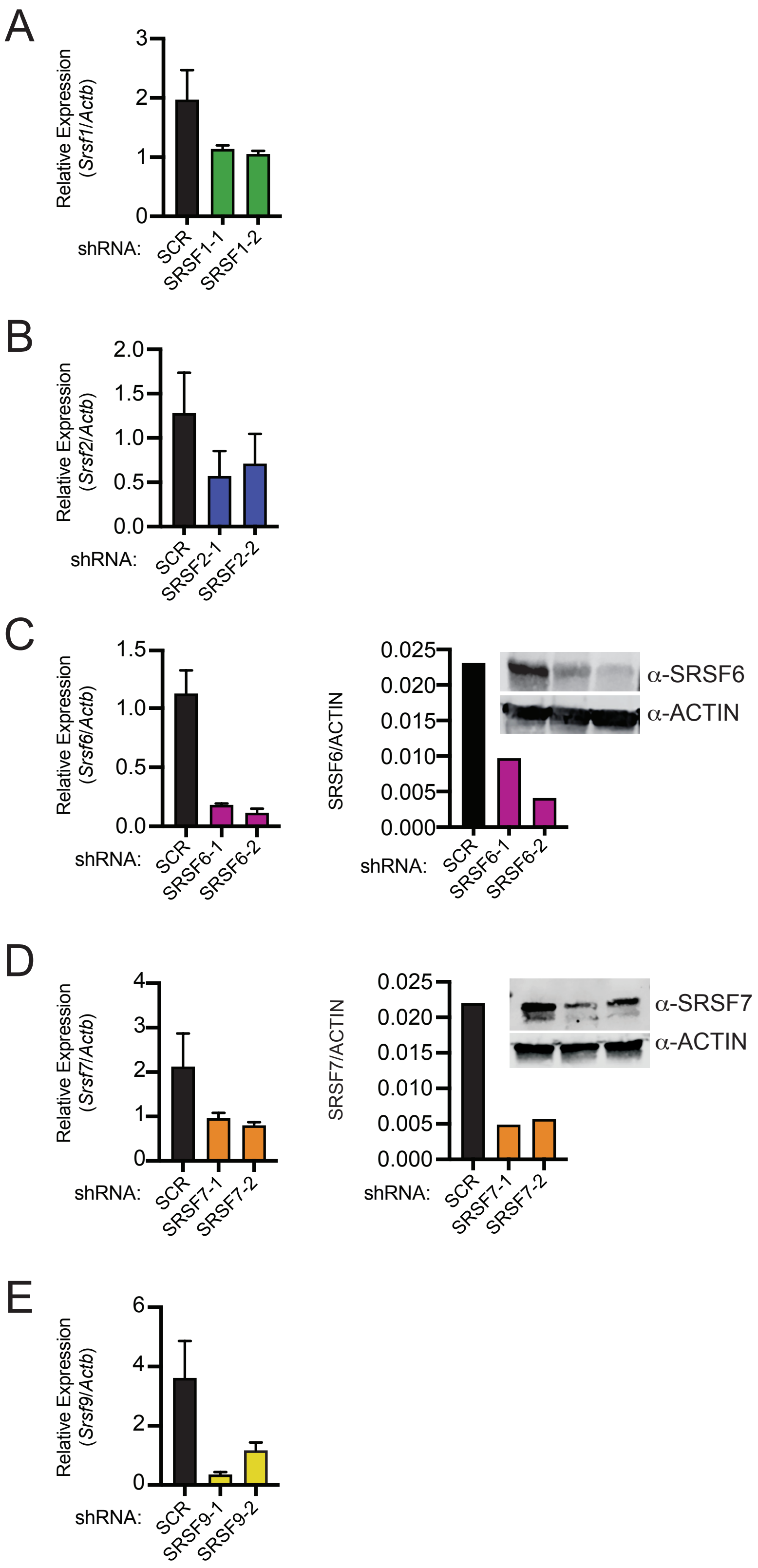

Figure S3: (A-E) Knockdown efficiency of SRSF RAW 264.7 macrophages by RT-qPCR (SRSF1, 2, 6 7, 9) and immunoblot (SRSF6, 7). For all RT-qPCRs, values are the mean of 3 biological replicates, error bars represent standard deviation, and immunoblots are representative of 2 or more independent experiments.

Figure S3

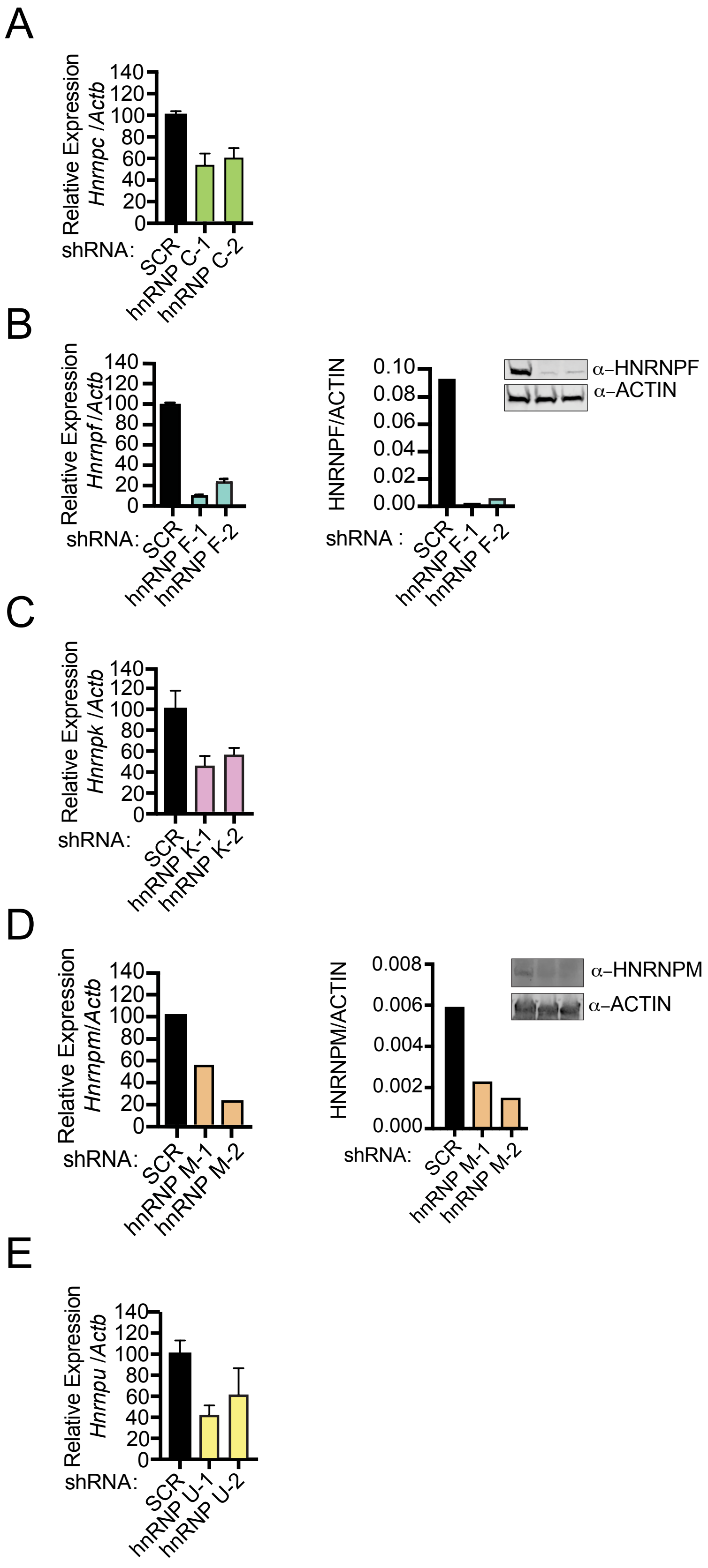

<u>Figure S4</u>: (A-E) Knockdown efficiency of hnRNP RAW 264.7 macrophages by RT-qPCR (hnRNP C, F, K, M, U) and immunoblot (hnRNP F, hnRNP M). For all RT-qPCRs, values are the mean of 3 biological replicates, error bars represent standard deviation, and immunoblots are representative of 2 or more independent experiments.

Figure S4

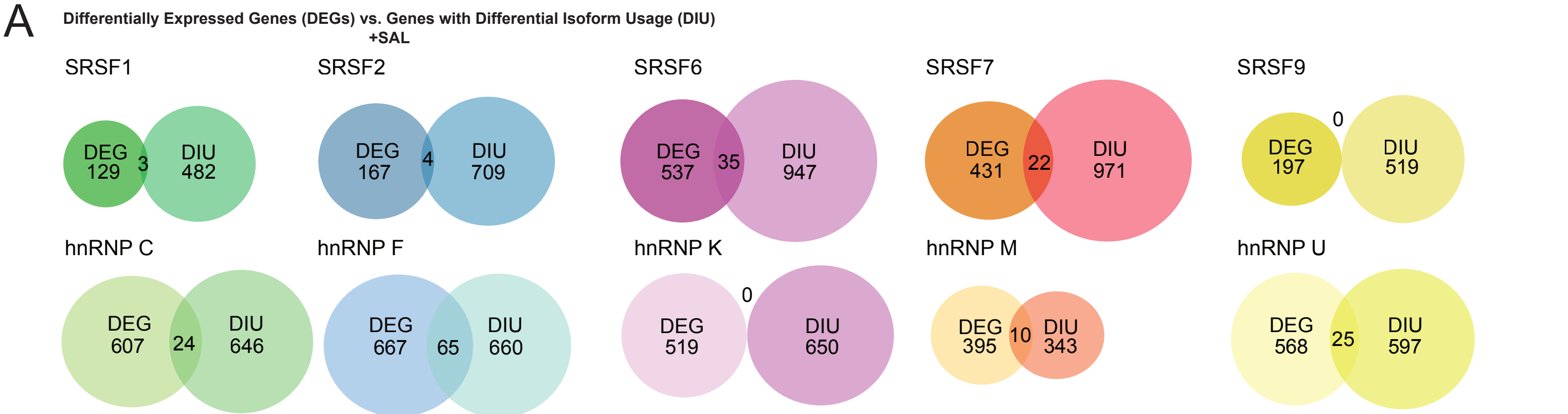

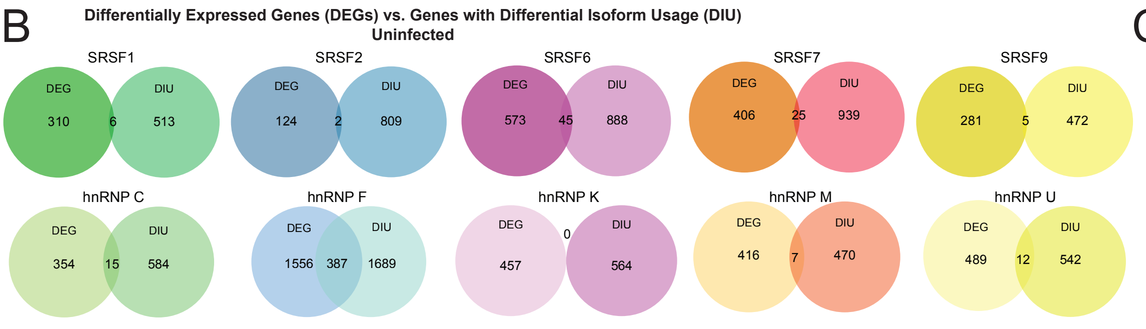

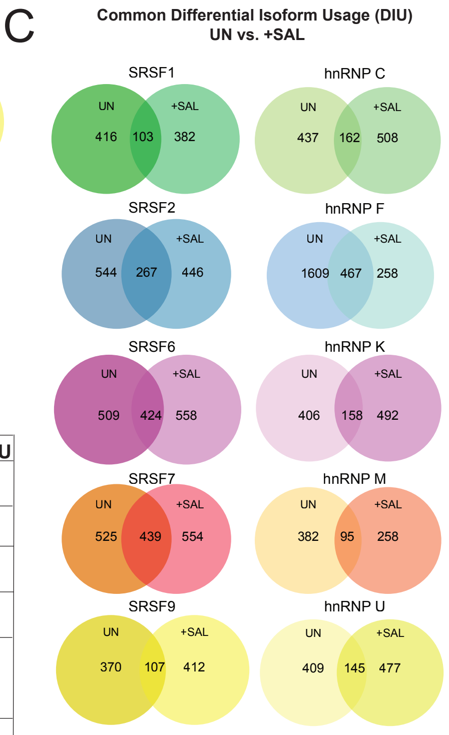

D

+Salmonella

	SRSF1	SRSF2	SRSF6	SRSF7	SRSF9	hnRNP C	hnRNP F	hnRNP K	hnRNP M	hnRNP U
Protein modification	*Senp7*	*Senp1*<br>*Senp7*	*Senp7*	*Dcun1d2*					*Ubqln1*	*Trim3*
Cell adhesion/migration	*Nav2*	*Cd44*	*Cd37*	*Afdn*			*Cd40*	*Plxnc1*		
Metabolism		*Comt*<br>*Fam135a*	*Pisd-ps52*	*Clcn4*<br>*Loxl3*	*Nadsyn1*	*Nit1*	*Mmgt2*		*Ald3a2*	
Membrane receptors/ Intracellular signalling				*Wnk1*		*Lrp8*	*Ikbke*<br>*Il2rg*	*Lrp8*		
Intracellular trafficking		*Nbr1*	*Rapgef1*<br>*Apls2*<br>*Pitpnb*	*Erc1*			*Arhgef1*<br>*Exoc7*<br>*Pex2*<br>*Snx11*	*Arhgap30*	*Ktn1*<br>*Xpo6*<br>*Atxn2*	*Sept9*
Chromatin factors Transcription/ Gene expression		*Ptpb2*<br>*Zscan26*	*Nfatc1*<br>*Baz2b*<br>*H2-Bl*	*Brd1*<br>*Hp1bp3*<br>*Sertad2*<br>*Zkscan17*	*E2f1*	*Emsy*<br>*Zfp426*	*Kdm7a*<br>*Nr2c2ap*<br>*Snapc4*<br>*Tfec*<br>*Tmpo*<br>*Zfp444*	*E2f1*<br>*Emsy*	*Asxl1*	
Chromosome biology/ Cell cycle		*Terf1*	*Cep295*<br>*Cspp1*<br>*Fance*<br>*Mdm4*	*Cspp1*<br>*Fance*<br>*Mphosph9*<br>*Ncapg2*<br>*Sapcd2*			*Csnk1d*<br>*Cuedc2*<br>*Numa1*	*Csf1*		
Other	*Xiap*		*Ints10*<br>*Lix1*<br>*Dnajb6*<br>*Ppcdc*	*Dop1a*<br>*Lrif1*<br>*Prr14l*<br>*Pphln1*<br>*Slc25a36*<br>*Tuft1*		*Nckap5l*	*Dnase1l1*<br>*Fmnl1*<br>*Plpp2*<br>*Tarbp2*<br>*Tmt2a*<br>*Slc16a3*<br>*Slc29a1*<br>*Zmiz2*	*Tmem229b*		

Intron Retention
Exon Skipping
Alternative 3'SS
Alternative 5'SS

Figure S5: (A) Overlap of DEGs (differentially expressed genes) with genes that have significant MAJIQ LSVs (differential isoform usage (DIU)) for SRSF (top) and hnRNP (bottom) knockdown RAW 264.7 macrophages vs. SCR controls in *Salmonella*-infected cells. DEG p<0.05; LSV PSI (PSI ≥10%). (B) Overlap of DEGs with genes that have significant MAJIQ LSVs for SRSF (top) and hnRNP (bottom) knockdown RAW 264.7 macrophages vs. SCR controls in uninfected cells. DEG p<0.05; LSV PSI (PSI ≥10%). (C) Overlap of genes with LSVs in uninfected vs. *Salmonella*-infected SRSF (left) and hnRNP (right) knockdown RAW 264.7 macrophages vs. SCR controls (PSI ≥10%). (D) Table representing top MAJIQ LSVs for SRSF and hnRNP knockdown RAW 264.7 macrophages vs. SCR controls in Salmonella infected cells Confidence threshold 0.95 and LSV PSI (PSI ≥20%).

Figure S5

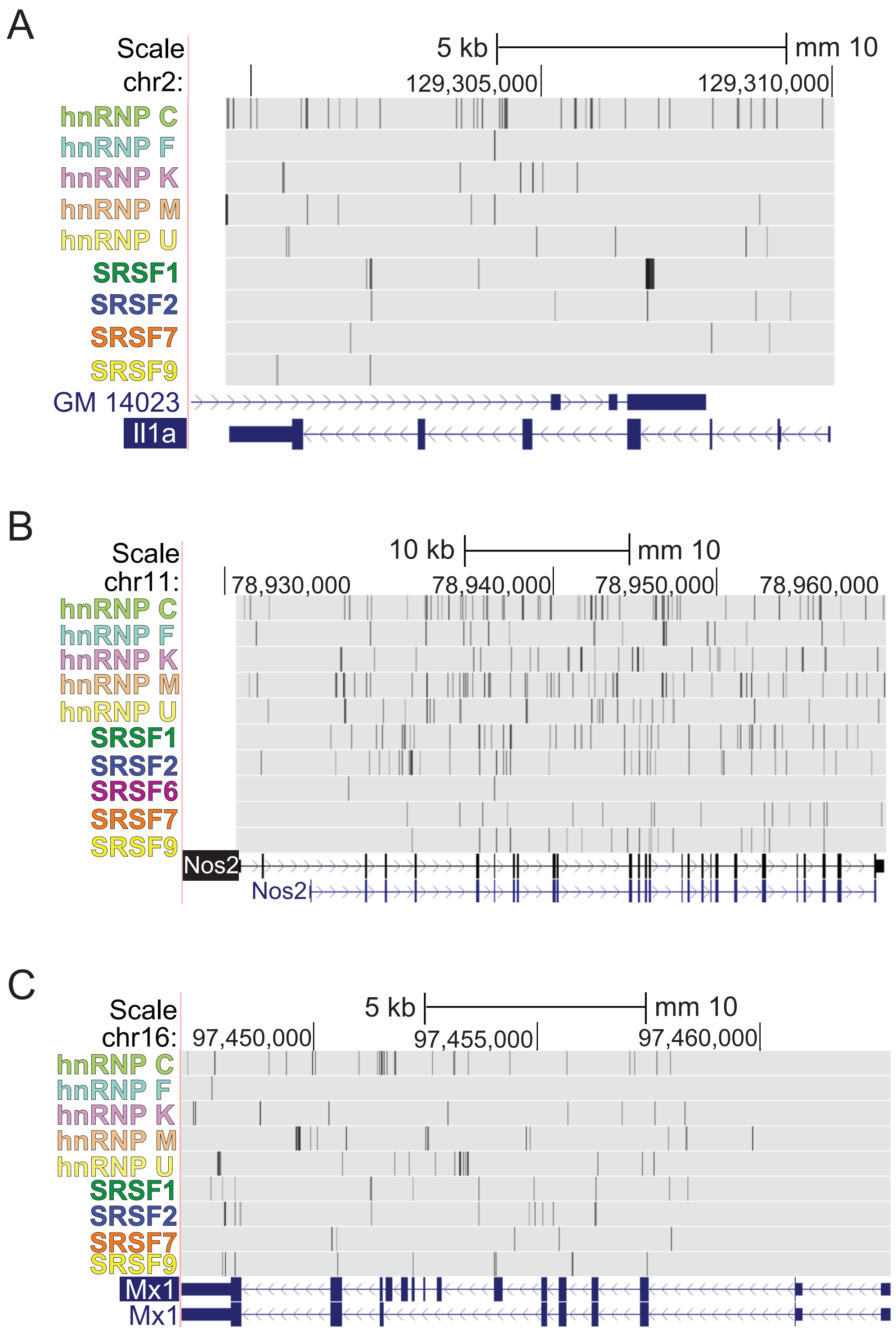

Figure S6: (A) Gene diagram of Il1a from UCSC genome browser with RBPmap (Paz et al., 2014) tracks for SRSF and hnRNP predicted binding sites. An SRSF/hnRNP track was included if the factor had one or more predicted binding sites in the gene queried. Mouse genome (GRCm38/mm10); high stringency; conservation filter applied in RBPmap). (B) As in (A) but for Nos2. (C) As in (A) but for Mx1.

Figure S6

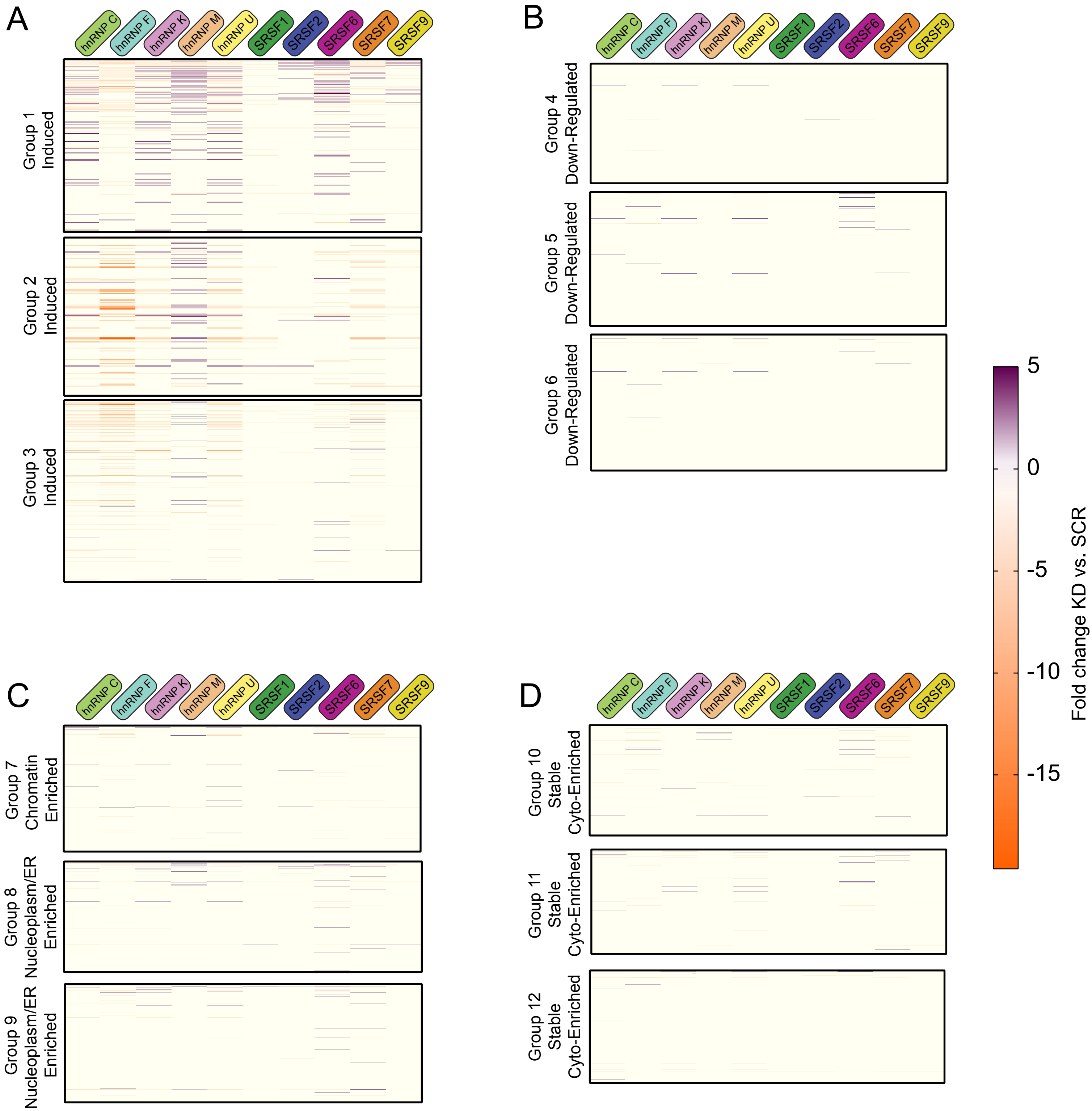

<u>Figure S7</u>: Fold change of SR/hnRNP DEGs in *Salmonella*-infected RAW 264.7 macrophages compared to SCR controls with all genes (RefSeq genes exceeding 400-bp in length with an RPKM of at least 1 in at least one sample) categorized on basis of expression kinetics and subcellular accumulation in chromatin, nucleoplasm, and cytoplasm fractions as defined by Bhatt et al., 2012. (A) Groups 1-3 contain genes induced by Lipid A treatment. (B) Groups 4-6 contain genes downregulated by Lipid A treatment. (C) Groups 7-9 contain transcripts enriched in a particular cellular compartment. (D) Groups 10-12 contain genes enriched in the cytoplasm.

Figure S7
